# Synthesis of Carlina Oxide Analogues and Evaluation of Their Insecticidal Efficacy and Cytotoxicity

**DOI:** 10.1021/acs.jnatprod.3c00137

**Published:** 2023-05-12

**Authors:** Eleonora Spinozzi, Marta Ferrati, Cecilia Baldassarri, Filippo Maggi, Roman Pavela, Giovanni Benelli, Cristina Aguzzi, Laura Zeppa, Loredana Cappellacci, Alessandro Palmieri, Riccardo Petrelli

**Affiliations:** † Chemistry Interdisciplinary Project (ChIP), School of Pharmacy, University of Camerino, Via Madonna delle Carceri, 62032 Camerino, Italy; ‡ Crop Research Institute, Drnovska 507, 161 06 Prague 6, Czech Republic; § Department of Plant Protection, Czech University of Life Sciences Prague, Kamycka 129, 165 00 Praha 6, Suchdol, Czech Republic; ∥ Department of Agriculture, Food and Environment, University of Pisa, Via del Borghetto 80, 56124 Pisa, Italy; ⊥ School of Pharmacy, University of Camerino, Via Madonna delle Carceri 9/C, 62032 Camerino, Italy; # School of Science and Technology, Chemistry Division, University of Camerino, Via Madonna delle Carceri, 62032 Camerino, Italy

## Abstract

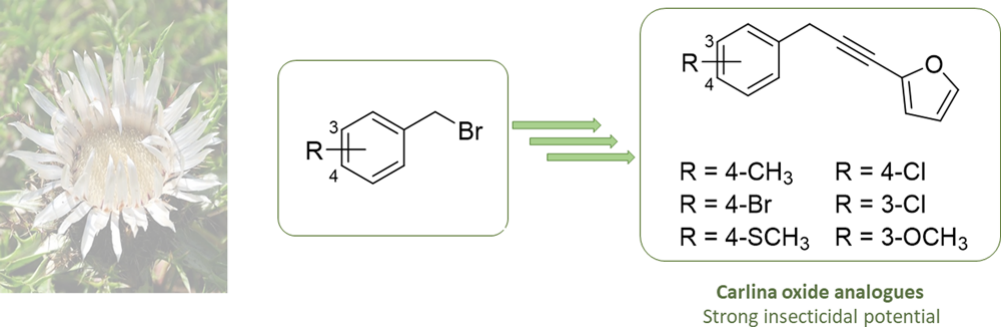

Compounds isolated from botanical sources represent innovative and promising alternatives to conventional insecticides. Carlina oxide is a compound isolated from *Carlina acaulis* L. (Asteraceae) essential oil (EO) with great potential as bioinsecticide, being effective on various arthropod vectors and agricultural pests, with moderate toxicity on non-target species. Since the production from the wild source is limited, there is the need of exploring new synthetic routes for obtaining this compound and analogues with improved bioactivity and lower toxicity. Herein, the chemical synthesis of carlina oxide analogues was developed. Their insecticidal activity was assessed on the vectors *Musca domestica* L. and *Culex quinquefasciatus* Say, and their cytotoxicity was evaluated on a human keratinocyte cell line (HaCaT). The compounds’ activity was compared with that of the natural counterparts EO and carlina oxide. In housefly tests, the analogues were comparably effective to purified carlina oxide. In *Cx. quinquefasciatus* assays, the *meta*-chloro analogue provided a significantly higher efficacy (LC_50_ of 0.71 μg mL^–1^) than the EO and carlina oxide (LC_50_ 1.21 and 1.31 μg mL^–1^, respectively) and a better safety profile than carlina oxide on keratinocytes. Overall, this study can open the way to an agrochemical production of carlina oxide analogues employable as nature-inspired insecticides.

The use of conventional insecticides has an enormous impact, boosting food production and contributing significantly to the improvement of human health, including the reduction of the onset of vector-borne diseases.^1,2^ However, the misuse and overuse of insecticides led to several negative consequences such as the accumulation in food, water, and soil, the development of pesticide resistance, and nontarget effects on human health and the ecosystem.^3−6^ In this context, botanical insecticides represent innovative and safe alternatives to conventional products due to their promising efficacy on a wide spectrum of vectors and agricultural pests and their moderate to low impact on the environment, as well as on human and animal health.^7−9^ However, the limited supply of the raw material from botanical sources may lead to exploring alternative routes for obtaining these insecticidal agents. *Carlina acaulis* L. is a medicinal plant belonging to the Asteraceae (Compositae) family and native to the calcareous soils of southern and central Europe^10^ with documented biological activities.^11−14^ Its root essential oil (EO) is characterized by the predominance (>95%) of 2-(3-phenylprop-1-ynyl)furan, commonly known as carlina oxide (**1**).^15^
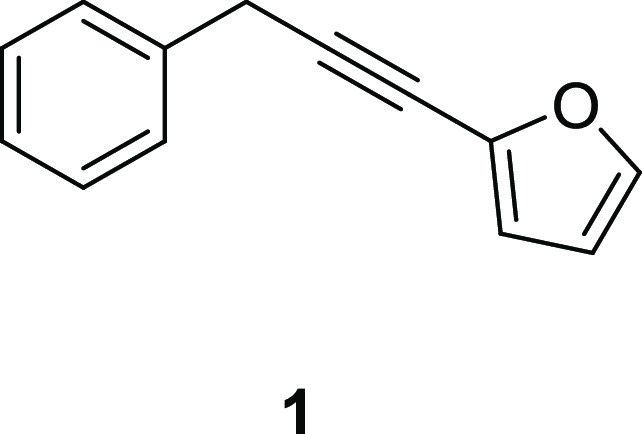


This compound (**1**) belongs to the class of polyacetylenes, which are well recognized as phytoalexins, i.e., defense substances produced by plants in response to living microorganisms, products of microbial origin, and environmental stress such as UV light exposure and cold.^16^*C. acaulis* EO, carlina oxide, and formulations encapsulating these products have been tested against vectors (*Culex quinquefasciatus* Say and *Musca domestica* L.), agricultural pests (*Lobesia botrana* (Denis & Schiffermüller), *Bactrocera oleae* (Rossi), *Ceratitis capitata* (Wiedemann), and *Meloidogyne incognita* (Kofoid & White)), and stored-products pests (*Acarus siro* L., *Alphitobius diaperinus* (Panzer), *Oryzaephilus surinamensis* L., *Prostephanus truncatus* (Horn), *Rhyzopertha dominica* (F.), *Sitophilus oryzae* L., *Tribolium confusum* Jacquelin du Val, *Tribolium castaneum* (Herbst), *Tenebrio molitor* L., and *Trogoderma granarium* Everts.), showing noteworthy results.^17−20^ The above-mentioned studies also demonstrated the limited toxicity of *C. acaulis* EO on nontarget species, as well as its promising safety profile in terms of LD_50_ and IC_50_ values determined on rats and human cells, respectively.^18,21,22^ Since carlina oxide is responsible for the *C. acaulis* EO insecticidal potential^20^ and given the unlikely possibility of obtaining it in a scalable manner from the natural source, this research aimed at the production of differently functionalized carlina oxide analogues through a three-step synthetic approach and the evaluation of their insecticidal activity against the adults of *M. domestica*, an ubiquitous fly pest and vector of several pathogens,^23^ and *Cx. quinquefasciatus* 3rd instar larvae, a lymphatic filariasis and arbovirus vector.^24,25^ The cytotoxicity of these analogues was also evaluated on immortalized human keratinocytes (HaCaT) and compared with that of their natural counterparts EO and carlina oxide.

## Results and Discussion

### Synthesis of Carlina Oxide Analogues

**Scheme 1:**
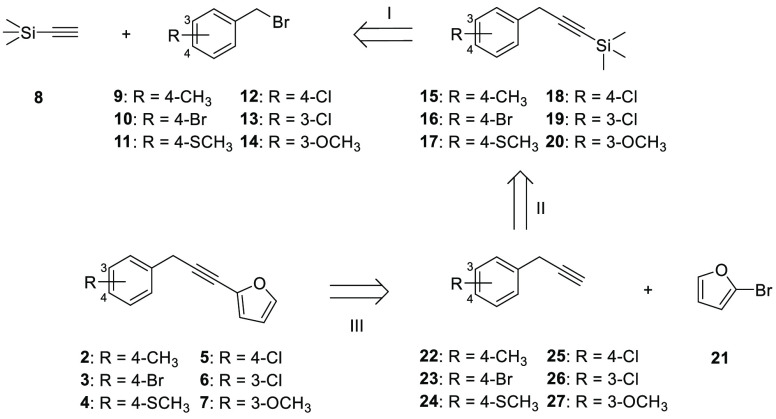
Retrosynthetic Analysis of the Synthetic Approach for Carlina Oxide Analogues

Since the carlina oxide scaffold resulted to be a promising lead compound for the development of new botanical insecticides, we synthesized a new series of carlina oxide analogues differently substituted on the benzyl moiety to evaluate their insecticidal activity. The synthetic protocol was developed using a retrosynthetic approach (Scheme 1) employing commercially available benzyl bromide substrates.

**Scheme 2:**
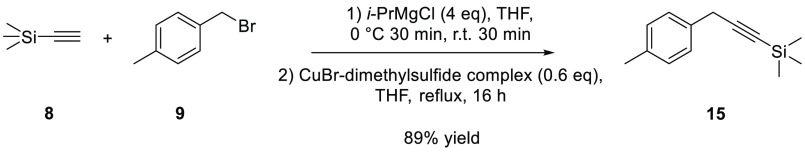
Substitution Reaction of Ethynyltrimethylsilane on 4-Methylbenzyl Bromide

The developed process involved three steps: (I) the substitution reaction of ethynyltrimethylsilane on the benzyl bromide substrates, (II) the deprotection of the terminal C–H of the alkynes, and (III) the Sonogashira coupling between the differently substituted terminal alkynes and 2-bromofuran. The model substrate used for the assessment of this synthetic approach was 4-methylbenzyl bromide (compound **9**). Inspired by the work of Hameury et al.,^26^ the reaction of **8** with **9** (Scheme 2) was investigated using initially ethylmagnesium bromide (EtMgBr) as base and copper bromide (CuBr) as catalyst, but no reactivity was observed. After different attempts, characterized by the change of the base or of the catalyst, the use of isopropylmagnesium chloride (*i*-PrMgCl) and of CuBr-dimethylsulfide complex in tetrahydrofuran (THF) for 16 h led to **15** formation in 89% yield (Scheme 2).

**Scheme 3:**
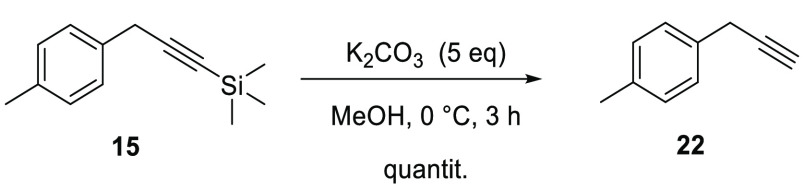
Synthesis of the Alkyne **22** quantit., quantitative yield.

At this point, following the study of Konno et al.,^27^ tetrabutylammonium fluoride (TBAF) was initially chosen as the C–H bond deprotecting agent for the synthesis of **22**, and several optimization efforts were conducted for the achievement of the optimal reaction conditions. Unfortunately, all those efforts have not paid off in terms of product formation. Thus, following the work of Louvel et al.,^28^ this step was performed using K_2_CO_3_ in MeOH for 3 h at 0 °C, obtaining a quantitative yield (Scheme 3).

**Scheme 4:**
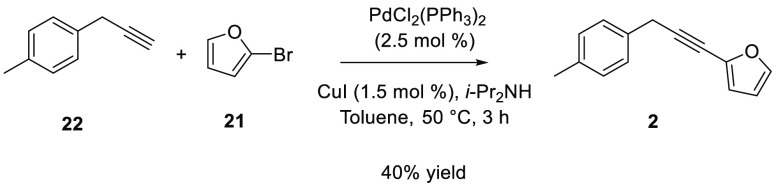
Synthesis of Compound **2**

Finally, for the synthesis of compound **2** a Sonogashira coupling between **22** and 2-bromofuran (**21**) was performed according to the procedure previously reported by Tomas-Mendivil et al.,^29^ using palladium(II)bis(triphenylphosphine) dichloride (PdCl_2_(PPh_3_)_2_), copper iodide (CuI), and diisopropylamine (*i*-Pr_2_NH) in toluene at 50 °C for 3 h. This step led to product formation in 40% yield (Scheme 4).

**Scheme 5:**
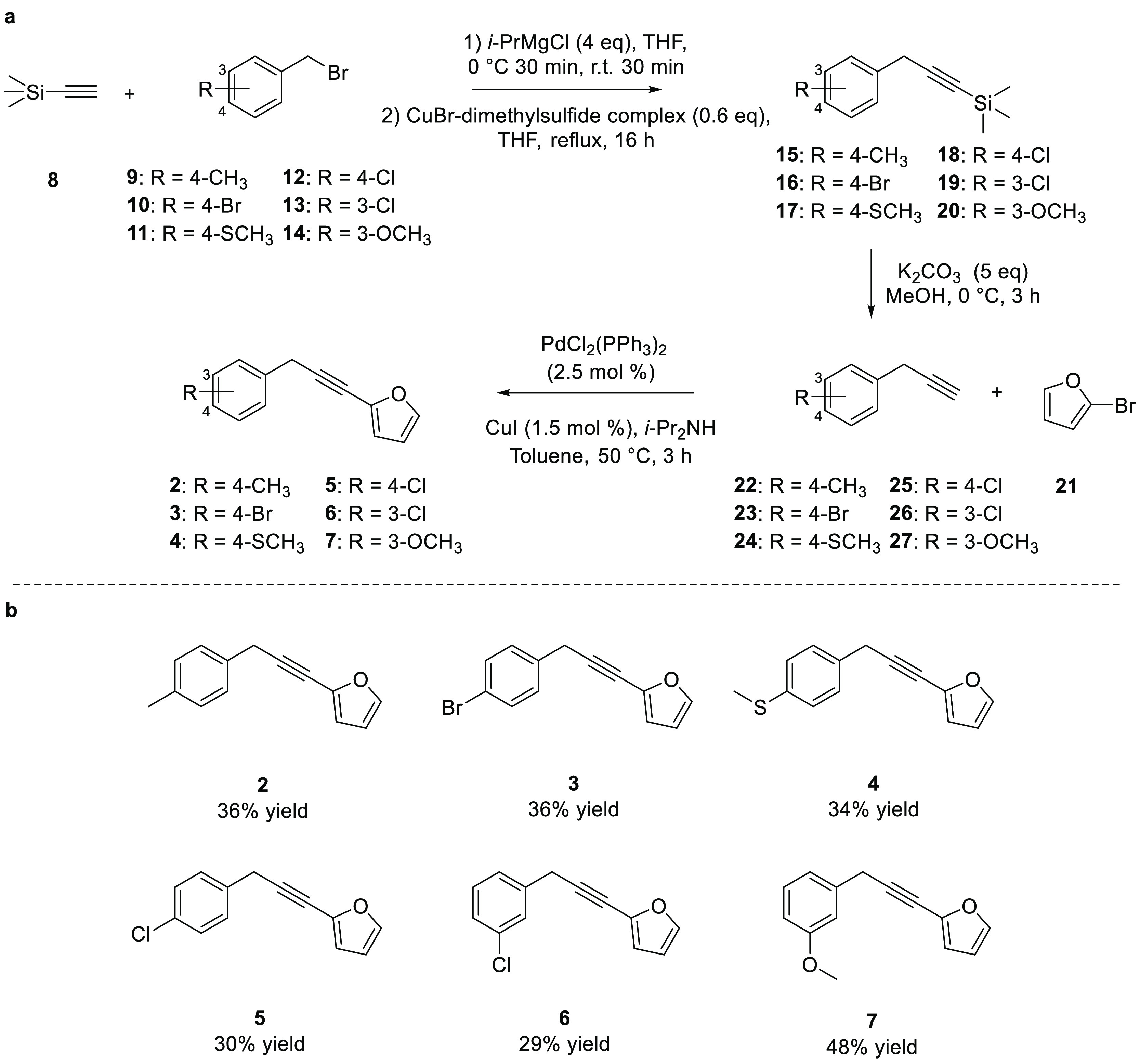
General Synthetic Protocol Developed for Carlina Oxide Analogues (a); Chemical Structures and Yield of Novel Carlina Oxide Analogues **2**–**7** (b)

This synthetic protocol developed for the synthesis of compound **2** was applied to other substituted benzyl bromides to obtain compounds **3**–**7** in different overall yields ranging from 29% to 48% (Scheme 5).

The *ortho*-chloro analogue synthesis was also attempted, but the isolation of the final product was not achieved. Detailed chemical procedures for the preparation of the presented compounds and the full characterization data are reported in the Experimental Section.

Chemical modifications of the carlina oxide scaffold were previously performed by Mami et al.^30^ The authors developed a hemisynthesis to produce a series of carlina oxide analogues by functionalization of the polyacetylene structure on the furan ring, with yields ranging from 17% to 30%. Therefore, we developed a novel and simple synthetic approach to produce carlina oxide analogues, and six new prototypes were obtained over three steps. Even if an overall yield optimization is still needed for the above-presented study, the latter represents the starting point for the synthesis of other carlina oxide derivatives. Moreover, this preliminary study aimed at the exploration of carlina oxide derivatives, and further work is needed to better understand structure–activity relationship.

### *Carlina acaulis* Essential Oil Chemical Analysis

In this work, *C. acaulis* EO was used for comparative purposes. Therefore, for the sake of completeness, its chemical composition is also reported, with carlina oxide (97.8%) representing the predominant constituent (Section S4, Supporting Information). Other minor compounds (<1%) detected in the EO were benzaldehyde (0.9%), *ar*-curcumene (0.7%), *β*-sesquiphellandrene (0.2%), and *α*-zingiberene (0.1%). This composition was fully consistent with those reported in the literature.^17,19,31^

### Topical Bioassays on *Musca domestica*

Carlina oxide analogues displayed high efficacy in terms of *M. domestica* female mortality. The estimated lethal doses are presented in Table 1. As indicated by the results, all the tested analogues showed an efficacy comparable to that of purified carlina oxide (**1**), for which the LD_50_ was estimated as 5.5 μg female^–1^ and LD_90_ as 11.5 μg female^–1^. Only **3**, **4**, and **5** provided significantly poorer efficacy; for the LD_50_(_90_) of other products, the CI_95_ values overlapped in at least one LD parameter, and thus they cannot be considered as significantly more effective, although, for example, the LD_50_ for **2** was estimated as 2.9 μg female^–1^.

**Table 1:** Mortality of *Musca domestica* Females after 24 h from Topical Application of the Tested Products

product tested	LD_50_ (μg female^**–**1^)	CI_95_	LD_90_ (μg female^**–**1^)	CI_95_	*χ*^*2*^^b^	*df*	*p*-value
*Carlina acaulis* EO^a^	5.3	3.2–7.1	11.7	8.9.21.8	6.451	3	0.521
Carlina oxide (**1**)	5.5	3.3–7.5	11.5	8.6–18.7	3.562	3	0.128
**2**	2.9	1.3–5.7	16.3	12.7–25.8	3.502	3	0.321
**3**	10.1	8.1–15.6	46.5	35.8–56.2	1.835	3	0.607
**4**	9.7	7.2–12.9	55.1	38.7–65.9	4.218	4	0.377
**5**	11.6	10.3–13.1	24.5	20.4–32.1	1.861	3	0.601
**6**	5.1	4.1–6.2	18.4	16.2–18.5	3.814	3	0.282
**7**	4.7	3.8–5.8	15.8	12.3–23.7	1.262	3	0.531

a EO, essential oil. LD_50_ and LD_90_ values in μg female^–1^ and CI_95_ are 95% confidence intervals; products’ activity is considered significantly different when the 95% CI fail to overlap.

b Chi-square value, not significant (ns, *p* > 0.05) level.

Acute toxicity on *Culex quinquefasciatus* larvae. The lethal concentrations estimated for *Cx. quinquefasciatus* are reported in Table 2. All the tested analogues showed very promising mortality on mosquito larvae. Nevertheless, based on LC_50_(_90_) comparison, **6** was found to provide a remarkably better efficacy, exhibiting a significantly lower lethal concentration (LC_50_(_90_) = 0.71(0.85) μg mL^–1^) compared with the EO and carlina oxide (**1**), which showed LC_50_(_90_) 1.21(2.28) μg mL^–1^ and 1.31(2.31) μg mL^–1^, respectively.

**Table 2:** Mortality of *Culex quinquefasciatus* Larvae (3rd Instar) after 24 h of Exposure to the Tested Products

product tested	LC_50_ (μg mL^**–**1^)	CI_95_	LC_90_ (μg mL^**–**1^)	CI_95_	*χ*^2^^b^	*df*	*p*-level
*Carlina acaulis* EO^a^	1.21	1.12–1.31	2.28	2.27–2.59	1.721	4	0.786
Carlina oxide (**1**)	1.31	1.12–1.47	2.31	1.96–3.05	7.869	5	0.163
**2**	2.25	1.47–2.69	3.61	2.74–6.41	4.446	3	0.188
**3**	1.44	1.23–1.86	2.41	2.11–3.28	3.852	3	0.421
**4**	1.21	1.11–1.31	2.36	2.13–2.67	3.251	5	0.128
**5**	1.11	0.93–1.23	2.12	1.95–2.23	2.529	4	0.896
**6**	0.71	0.55–0.78	0.85	0.81–1.25	2.189	3	0.334
**7**	0.73	0.62–1.11	1.43	1.22–2.02	3.181	3	0.364

a EO, essential oil. LC_50_ and LC_90_ values in μg mL^–1^ and CI_95_ are 95% confidence intervals; products’ activity is considered significantly different when the 95% CI fail to overlap.

b Chi-square value, not significant (ns, *p* > 0.05) level.

The introduction of a substituent on the benzyl ring differently modulated the biological activity of the analogues. In detail, the *meta*-substitution (compounds **6** and **7**) led to the best LC_50_ values. This kind of substitution is probably crucial for the promising larvicidal activity on *Cx*. *quinquefasciatus*, and the mechanism of action should be further investigated. The best acute toxicity was observed by the *meta*-chloro analogue. Indeed, the chlorine substituent has been shown to be essential for several insecticidal compounds like dichlorodiphenyltrichloroethane (DDT), but also for natural products used as antibiotics and antitumor agents, such as clindamycin and vancomycin^32,33^ or cryptophycin and clavulone.^34,35^ The presence of chlorine on an aromatic moiety generally causes an increase of lipophilicity, nonbonding interactions with the binding sites, prevention of metabolic hydroxylation at that position, and increase of the electrophilicity of proximate parts of the molecule due to its electronegativity.^36^ However, also the *meta*-methoxy analogue showed a good toxicity on *Cx. quinquefasciatus* larvae. The presence of the methoxy group in the *meta*-position of compound **7** could enhance its lipophilicity and hence increase membrane permeability.^37^ However, this hypothesis should be further investigated.

### Sublethal Effect against *Culex quinquefasciatus* Larvae

Exposing mosquito larvae to the LC_30_ estimated through acute toxicity assays achieved subsequent significant mortality on *Cx. quinquefasciatus* (except for compound **7**) despite a relatively short exposure period (24 h). Compared with untreated larvae, a significantly lower percentage of adults emerged from insecticide-exposed larvae (Table 3). Nevertheless, differences could be observed between the individual tested products. Compound **3** provided the highest efficacy, causing mortality of 71% larvae and hatching of only 28.7% adults upon application at the concentration of 1 μg mL^–1^. This result was identical to that observed for the EO and even significantly better compared with carlina oxide (**1**).

**Table 3:** Being Exposed for 24 h to the LC_30_ of *Carlina acaulis* Essential Oil, Carlina Oxide, and Its Synthesized Analogues Affected *Culex quinquefasciatus* Larval and Pupal Mortality as Well as the Percentage of Successfully Emerged Adults

product tested	concentration (μL L^**–**1^)	larval mortality (% ± SE)	pupal mortality (% ± SE)	emerged adults (% ± SE)
*Carlina acaulis* EO	0.9	79.0 ± 5.7^e^	0.7 ± 0.9	20.3 ± 5.2^a^
Carlina oxide (**1**)	0.8	58.3 ±7.4^d^	0.03 ± 0.5	41.3 ± 6.9^b^
**2**	1.5	53.0 ± 2.2^d^	0.0 ± 0.0	47.0 ± 2.2^b^
**3**	1.0	71.0 ± 5.0^e^	0.3 ± 0.5	28.7 ± 4.7^a^
**4**	0.8	32.7 ± 3.4^c^	0.7 ± 0.5	66.7 ± 3.9^c^
**5**	0.8	33.8 ± 3.1^c^	0.5 ± 0.2	69.5 ± 3.2^c^
**6**	0.5	39.0 ± 8.6^c^	1.3 ± 0.4	59.7 ± 8.2^c^
**7**	0.5	8.7 ± 2.4^b^	0.3 ± 0.5	91.0 ± 2.8^d^
Control		3.0 ± 2.2^a^	0.0 ± 0.0	97.0 ± 2.2^d^
ANOVA *F*_8, 32_, *p*-value^a^		98.3; 0.000	ns	184.3; 0.000

a ANOVA parameters. In the same column, means followed by different letters are significantly different (ANOVA, Tukey’s HSD test, *p* < 0.05). EO = essential oil; ns = not significant (*p* > 0.05).

The results reported above pointed out a better efficacy of compound **3**, bearing a bromine atom in the *para*-position, with respect to the *meta*-chloro-substituted analogue (**6**) that was the most active in the acute toxicity assays.

### Larval Mortality Dynamics in Time, upon Application of LC_90_

The dynamics of *Cx. quinquefasciatus* larval mortality upon application of a concentration corresponding to the LC_90_ are presented in Table 4. No or almost no mortality was observed for the first 8 h from the application. A dynamic increase in larval mortality was observed only 12 h from exposure, and fatal mortality occurred within 24 h from application in all variants except **6**, which caused 86.7% mortality, and **3**, which led to 55.1% larval mortality. Compound **2** showed the highest dynamics, exhibiting the same course of the mortality increase rate as the EO, although it should be noted that a much higher applied concentration was used, i.e., 3.6 μg mL^–1^, while the EO was applied at the concentration of 2.3 μg mL^–1^.

**Table 4:** *Culex quinquefasciatus* Larval Mortality over Time When Exposed to the Estimated LC_90_ Values of *Carlina acaulis* Essential Oil, Carlina Oxide, and Its Synthesized Analogues

	time					
product concentration (μL L^**–**1^)	4 h	8 h	12 h	16 h	20 h	24 h
*Carlina acaulis* EO (2.3)	0.0 ± 0.0	0.0 ± 0.0^a^	78.6 ± 4.1^f^	100 ± 0.0^e^	100 ± 0.0^d^	100 ± 0.0^d^
Carlina oxide (**1**) (2.3)	0.0 ± 0.0	0.0 ± 0.0^a^	62.9 ± 2.8^g^	96.7 ± 2.4^e^	100 ± 0.0^d^	100 ± 0.0^d^
**2** (3.6)	0.0 ± 0.0	0.0 ± 0.0^a^	71.9 ± 4.1^f^	100 ± 0.0^e^	100 ± 0.0^d^	100 ± 0.0^d^
**3** (2.4)	0.0 ± 0.0	5.2 ± 1.2^b^	28.9 ± 2.5^d^	45.0 ± 4.1^b^	46.7 ± 4.1^b^	55.1 ± 4.1^b^
**4** (2.4)	0.0 ± 0.0	0.0 ± 0.0^a^	12.8 ± 1.2^b^	93.3 ± 6.2^e^	98.3 ± 2.4^d^	100 ± 0.0^d^
**5** (2.1)	0.0 ± 0.0	0.0 ± 0.0^a^	43.8 ± 3.5^e^	78.7 ± 4.7^c^	95.2 ± 1.5^d^	100 ± 0.0^d^
**6** (1.0)	0.0 ± 0.0	0.0 ± 0.0^a^	18.9 ± 2.1^c^	75.0 ± 8.2^c^	81.7 ± 2.4^c^	86.7 ± 6.1^c^
**7** (1.5)	0.0 ± 0.0	0.0 ± 0.0^a^	45.7 ± 3.2^e^	86.7 ± 4.7^c^	98.3 ± 1.2^d^	100 ± 0.0^d^
Control	0.0 ± 0.0	0.0 ± 0.0^a^	0.0 ± 0.0^a^	0.0 ± 0.0^a^	0.0 ± 0.0^a^	0.0 ± 0.0^a^
ANOVA *F*_8, 32_, *p*-value^a^	ns	7.0; 0.005	395.6; 0.000	874.2; 0.000	1528.2; 0.000	1335.3; 0.000

a ANOVA parameters. In the same column, means followed by different letters are significantly different (ANOVA, Tukey’s HSD test, *p* < 0.05). EO = essential oil; ns = not significant (*p* > 0.05).

Conversely to the acute toxicity results, herein the less active analogues after 24 h were **6** (86.7% mortality) and **3** (55.1% mortality). These analogues bear a chlorine and a bromine atom in the *meta*- and *para*-positions, respectively. These results suggest that the presence of an electronegative atom on the benzyl moiety probably does not significantly affect the mosquito larval mortality over time when exposed to concentrations corresponding to LC_90_ values. Further experiments on the reported synthesized compounds elucidating their efficacy on different targets as well as their possible modes of action are needed.

### Cell Viability Assay

**Figure 1:**
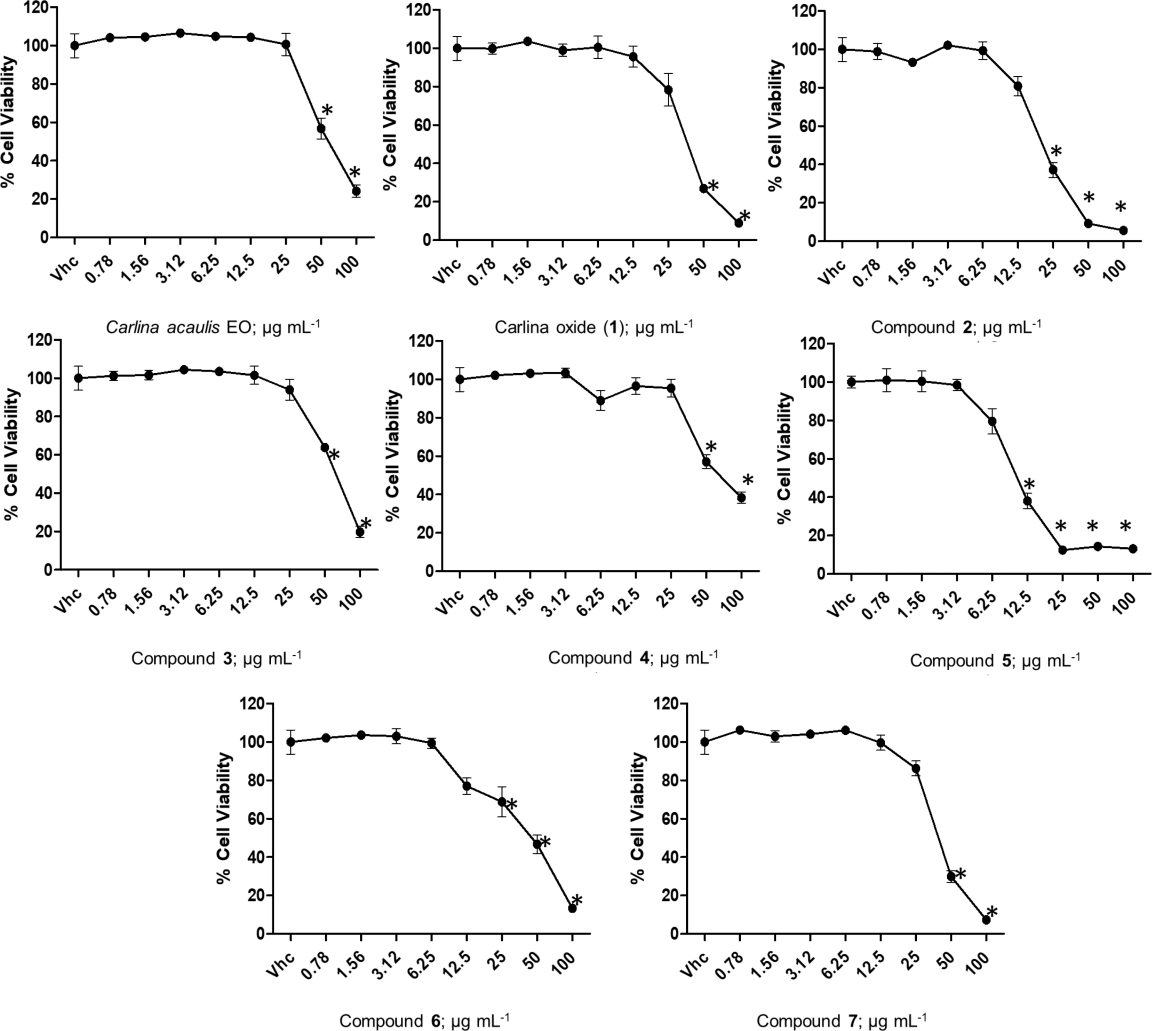
Cell viability was determined in the HaCaT cell line by the MTT assay after treatment for 72 h with different concentrations of *Carlina acaulis* Essential Oil, Carlina Oxide (**1**), and Carlina Oxide Analogues. Data are expressed as mean ± standard deviation (SD) of three separate experiments. *p < 0.05 vs vehicle (Vhc).

The cytotoxicity assay was performed to assess the safety profile of the synthesized analogues on immortalized human keratinocytes (HaCaT). Indeed, one of the main routes of exposure to insecticides is through the skin, and this represents a significant risk causing great concern in terms of operator safety.^38^ Results obtained from the cytotoxicity assay showed that compounds **2** and **5** induced a significant reduction in cell viability (HaCaT cell line) compared with carlina oxide (**1**) and *C. acaulis* EO, with IC_50_ values of 20.26 ± 1.2 and 9.18 ± 0.3 μg mL^–1^, respectively, compared with 34.85 ± 2.4 μg mL^–1^ for carlina oxide and 54.05 ± 5.0 μg mL^–1^ for the EO. Moreover, compound **7** showed a similar effect to carlina oxide (**1**) with an IC_50_ of 37.39 ± 2.8 μg mL^–1^. On the other hand, compounds **3** and **4**, with an IC_50_ of 60.38 ± 3.5 and 52.68 ± 3.7 μg mL^–1^, respectively, showed a better safety profile compared with carlina oxide (**1**), similar to that of EO (Table 5, Figure 1).

**Table 5:** *Carlina acaulis* Essential Oil, Carlina Oxide (**1**), and Carlina Oxide Analogues’ Cytotoxic Effects on HaCaT Cells

product tested	IC_50_ ± SD (μg mL^**–**1^)
*Carlina acaulis* EO^a^	54.05 ± 5.0^a^
Carlina oxide (**1**)	34.85 ± 2.4^b^
**2**	20.26 ± 1.2^d^
**3**	60.38 ± 3.5^c^
**4**	52.68 ± 3.7^a^
**5**	9.18 ± 0.3^e^
**6**	58.40 ± 3.1^c^
**7**	37.39 ± 2.8^b^

a EO, essential oil. Data shown are expressed as mean ± standard deviation (SD) of three separate experiments. IC_50_ ± SD within a column followed by the same letter do not differ significantly (ANOVA, Tukey’s HSD test, *p* ≤ 0.05).

Analogue **6** displayed the highest efficacy with respect to carlina oxide (**1**) in the acute toxicity assay on *Cx. quinquefasciatus*. It also showed a lower cellular toxicity (IC_50_ of 58.40 ± 3.1 μg mL^–1^) if compared with its precursor (IC_50_ of 34.85 ± 2.4 μg mL^–1^) and a similar toxicity to that of the EO (IC_50_ of 54.05 ± 5.0 μg mL^–1^). This result is highly encouraging, given the crucial importance of developing novel effective candidate insecticides with also a low cytotoxicity.

*C. acaulis* EO was previously tested on the primary human fibroblast cell line (NHFA12), showing a moderate toxicity (IC_50_ of 115.92 ± 6.1 μg mL^–1^). Moreover, it was also demonstrated that this toxicity became negligible when the EO was encapsulated into a microemulsion (ME) (IC_50_ of 5392.8 ± 315 μg mL^–1^).^22^ These results suggest that once encapsulated into nanocarriers (at 5–10% level), such as nanoemulsions, nanoparticles, or liposomes, carlina oxide analogues could be less cytotoxic according to the International Standard Organization (ISO) guidelines,^39^ where the reduction of cell viability should not exceed 70% at 100 ppm.^22^

The indiscriminate use of conventional insecticides causes an increase in arthropod pest and vector resistance along with negative effects on human and animal health and environmental pollution. Thus, the need for new, effective, and safe insecticides is growing. In this respect, botanicals represent a promising option to face these concerning issues. *C. acaulis* EO has already shown great potential against several insect pests and vectors. This study highlighted the possibility of synthesizing some carlina oxide analogues with better insecticidal activity than the natural polyacetylene. The *meta*-chloro substitution on the benzyl moiety led to improved mosquito larval toxicity and reduced cytotoxicity on human cells. On the other hand, most of the analogues, when used at their LC_90_, caused an almost complete mortality of mosquito larvae within 24 h and a similar efficacy in topical assays on housefly adults. In conclusion, further work is needed to better understand structure–activity relationships of carlina oxide derivatives as well as their mechanism of action in order to propose them as new insecticide leads to be exploited by agrochemical industries.

## Experimental Section

### General Experimental Procedures

All reagents and solvents were purchased from Merk KGaA (Darmstadt, Germany) and used without additional purification, except THF (freshly distilled over metallic sodium) and toluene (dried over 3 Å molecular sieves). ^1^H (400 or 500 MHz) and ^13^C (100 MHz) spectra were acquired on Varian Mercury 400 and 500 (Varian, Inc., Palo Alto, CA, USA). IR spectra (cm^–1^) were recorded with a PerkinElmer FT-IR spectrometer Spectrum Two UATR (PerkinElmer, Inc., Waltham, MA, USA). GC-MS analysis of the synthesized products was performed using a Hewlett-Packard GC/MS 6890N working with the EI technique (70 eV). Compound purity was evaluated through GC-MS and NMR analyses and was >90% for all the compounds. Elemental analyses (C, H, N, S) were conducted using a Fisons Instruments EA-1108 CHNS-O elemental analyzer (Thermo Fisher Scientific Inc., Waltham, MA, USA).

### General Synthesis of Compounds **15**–**20**

Compounds **15**–**20** were synthesized according to the procedure of Hameury et al.^26^ with some modifications. *i*-PrMgCl (2 M in THF, 20.0 mmol, 4.0 equiv) was slowly added at 0 °C to a solution of ethynyltrimethylsilane (20.0 mmol, 4.0 equiv) in THF (10 mL). The reaction mixture was stirred for 30 min at 0 °C and for an additional 30 min at rt. Then, CuBr-dimethylsulfide complex (3 mmol, 0.6 equiv) was added all at once, and the mixture was stirred at rt for 30 min before the addition of substrates **9**–**14** (5.0 mmol). The reaction was refluxed for 16 h. After that time, the solution was cooled to rt and poured into a saturated aqueous solution of NH_4_Cl (200 mL). The aqueous phase was extracted with Et_2_O (2 × 250 mL), and the organic layers were washed with H_2_O (200 mL), dried over MgSO_4_, filtered, and concentrated. The crude products were purified by silica gel chromatography (100% *n*-hexane) to afford the products in different yields.

#### (3-(*p*-Tolyl)-prop-1-yn-1-yl)-trimethylsilane (**15**):

colorless oil (174 mg, 86% yield); IR (neat) 2959, 2176, 1514, 1418, 1249, 1030, 1020, 838, 793, 758, 638, 476 cm^–1^; NMR spectra were in accordance with data reported in the literature:^28^^1^H NMR (CDCl_3_, 400 MHz) δ 7.26–7.18 (m, 2H), 7.17–7.08 (m, 2H), 3.61 (s, 2H), 2.33 (s, 3H), 0.18 (s, 9H); ^13^C NMR (CDCl_3_, 100 MHz) δ 136.3, 133.5, 129.3, 127.9, 104.8, 86.7, 25.9, 21.2, 0.2; MS (EI) *m*/*z* = 202 (M^+^), 187 (100%), 172, 157, 128, 73; anal. C 77.14, H 9.01%, calcd for C_13_H_18_Si, C 77.16, H 8.97%.

#### (3-(4-Bromophenyl)-prop-1-yn-1-yl)-trimethylsilane (**16**):

colorless oil (216 mg, 81% yield); IR (neat) 2959, 2178, 1515, 1405, 1248, 1071, 1028, 1012, 841, 791, 759, 635, 474 cm^–1^; ^1^H NMR (CDCl_3_, 500 MHz) δ 7.46–7.42 (m, 2H), 7.24–7.19 (m, 2H,), 3.60 (s, 2H), 0.19 (s, 9H); ^13^C NMR (CDCl_3_, 100 MHz) δ 131.7, 129.8, 129.2, 128.4, 103.6, 87.6, 25.9, 21.2, 0.2; MS (EI) *m*/*z* = 267 (M^+^), 252 (100%), 222, 194, 172, 128, 73; anal. C 53.90, H 5.62%, calcd for C_12_H_15_BrSi, C 53.93, H 5.66%.

#### (3-(4-(Methylthio)phenyl)-prop-1-yn-1-yl)-trimethylsilane (**17**):

colorless oil (169 mg, 72% yield); IR (neat) 2958, 2175, 1492, 1404, 1248, 1091, 967, 908, 839, 794, 732, 642, 484 cm^–1^; ^1^H NMR (CDCl_3_, 500 MHz) δ 7.31–7.27 (m, 2H), 7.27–7.24 (m, 2H), 3.64 (s, 2H), 2.50 (s, 3H), 0.21 (s, 9H); ^13^C NMR (CDCl_3_, 100 MHz) δ 136.6, 133.6, 128.5, 127.3, 104.3, 87.1, 25.8, 16.4, 0.2; MS (EI) *m*/*z* = 234 (M^+^, 100%), 187, 159, 109, 73; anal. C 66.62, H 7.72, S 13.66%, calcd for C_13_H_18_SSi, C 66.60, H 7.74, S 13.68%.

#### (3-(4-Chlorophenyl)-prop-1-yn-1-yl)-trimethylsilane (**18**):

colorless oil (174 mg, 78% yield); IR (neat) 2963, 2165, 1498, 1400, 1204, 1037, 1018, 1009, 829, 801, 773, 622, 482 cm^–1^; NMR spectra were in accordance with data reported in the literature:^40^^1^H NMR (CDCl_3_, 500 MHz) δ 7.29–7.27 (m, 4H), 3.61 (s, 2H), 0.19 (s, 9H); ^13^C NMR (CDCl_3_, 100 MHz) δ 135.0, 130.5, 129.4, 128.7, 103.7, 87.5, 25.8, 0.2; MS (EI) *m*/*z* = 222 (M^+^), 207 (100%), 179, 128, 73; anal. C 64.68, H 6.80%, calcd for C_12_H_15_ClSi, C 64.69, H 6.79%.

#### (3-(3-Chlorophenyl)-prop-1-yn-1-yl)-trimethylsilane (**19**):

colorless oil (138 mg, 62% yield); IR (neat) 2959, 2178, 1431, 1250, 1075, 1029, 1010, 840, 759, 649, 474 cm^–1^; ^1^H NMR (CDCl_3_, 400 MHz) δ 7.38–7.32 (m, 1H), 7.25–7.14 (m, 3H), 3.63 (s, 2H), 0.20 (s, 9H); ^13^C NMR (CDCl_3_, 100 MHz) δ 138.5, 134.4, 129.8, 128.3, 127.0, 126.2, 103.3, 87.8, 26.0, 0.2; MS (EI) *m*/*z* = 222 (M^+^), 207 (100%), 179, 128, 73; anal. C 64.65, H 6.74%, calcd for C_12_H_15_ClSi, C 64.69, H 6.79%.

#### (3-(3-Methoxyphenyl)-prop-1-yn-1-yl)trimethylsilane (**20**):

yellow oil (181 mg, 83% yield); IR (neat) 2958, 2176, 1435, 1249, 1026, 1015, 840, 759, 650, 441 cm^–1^; ^1^H NMR (CDCl_3_, 400 MHz) δ 7.26–7.18 (m, 1H), 6.95–6.86 (m, 2H), 6.79–6.73 (m, 1H), 3.79 (s, 3H), 3.62 (s, 2H), 0.17 (s, 9H); ^13^C NMR (CDCl_3_, 100 MHz) δ 159.9, 138.0, 129.6, 120.4, 113.6, 112.3, 104.3, 87.2, 55.3, 26.3, 0.2; MS (EI) *m*/*z* = 218 (M^+^), 189 (100%), 173, 73; anal. C 71.53, H 8.29%, calcd for C_13_H_18_OSi, C 71.50, H 8.31%.

### Synthesis of Compounds **22**–**27** and **21**

The synthesis of compounds **22**–**27** was performed following the work of Louvel et al.^28^ Compound **21** was prepared according to the procedure previously reported by Gilman and Wright,^41^ and its IR, NMR, and MS spectra were in line with those reported.^41^

#### 1-Methyl-4-(prop-2-yn-1-yl)benzene (**22**):

colorless oil (130 mg, quantitative yield); IR (neat) 2256, 1489, 1157, 1125, 990, 915, 732, 660, 597, 493 cm^–1^; NMR spectra were in accordance with data reported in the literature:^42^^1^H NMR (CDCl_3_, 500 MHz) δ 7.44–7.38 (m, 2H), 7.33–7.28 (m, 2H), 3.74 (d, *J* = 2.7 Hz, 2H), 3.03 (s, 1H), 2.49 (s, 3H); ^13^C NMR (CDCl_3_, 100 MHz) δ 136.4, 135.4, 129.2, 128.4, 82.4, 70.3, 24.5, 21.2; MS (EI) *m*/*z* = 130 (M^+^), 105 (100%), 91; anal. C 92.25, H 7.71%, calcd for C_10_H_10_, C 92.26, H 7.74%.

#### 1-Bromo-4-(prop-2-yn-1-yl)benzene (**23**):

yellow oil (168 mg, 86% yield); IR (neat) 2256, 1488, 1220, 1157, 989, 910, 729, 652, 595, 490 cm^–1^; NMR spectra were in accordance with data reported in the literature:^43^^1^H NMR (CDCl_3_, 500 MHz) δ 7.47–7.43 (m, 2H), 7.25–7.22 (m, 2H), 3.56 (d, *J* = 2.7 Hz, 2H), 2.20 (t, *J* = 2.7 Hz, 1H); ^13^C NMR (CDCl_3_, 100 MHz) δ 135.3, 131.8, 129.8, 120.8, 81.4, 71.0, 24.5; MS (EI) *m*/*z* = 195 (M^+^), 115 (100%), 89, 63; anal. C 55.44, H 3.64%, calcd for C_9_H_7_Br, C 55.42, H 3.62%.

#### Methyl-(4-(prop-2-yn-1-yl)phenyl)sulfane (**24**):

yellow solid (153 mg, 94% yield); IR (neat) 2253, 1492, 1210, 1153, 985, 904, 727, 649, 592, 488 cm^–1^; NMR spectra were in accordance with data reported in the literature:^42^^1^H NMR (CDCl_3_, 500 MHz) δ 7.32–7.28 (m, 2H), 7.28–7.23 (m, 2H), 3.59 (d, *J* = 2.7 Hz, 2H), 2.50 (s, 3H), 2.21 (t, *J* = 2.7 Hz, 1H); ^13^C NMR (CDCl_3_, 100 MHz) δ 136.8, 133.3, 128.5, 127.3, 82.0, 70.6, 24.4, 16.3; MS (EI) *m*/*z* = 162 (M^+^), 147, 115 (100%), 89, 63; anal. C 73.04, H 6.18, S 19.73%, calcd for C_10_H_10_S, C 74.03, H 6.21, S 19.76%.

#### 1-Chloro-4-(prop-2-yn-1-yl)benzene (**25**):

yellow oil (122 mg, 81% yield); IR (neat) 2263, 1501, 1203, 1174, 995, 923, 756, 674, 640, 470 cm^–1^; NMR spectra were in accordance with data reported in the literature:^44^^1^H NMR (CDCl_3_, 500 MHz) δ 7.24–7.18 (m, 2H), 7.04–6.97 (m, 2H), 3.55 (d, *J* = 2.6 Hz, 2H), 2.17 (t, *J* = 2.8 Hz, 1H); ^13^C NMR (CDCl_3_, 100 MHz) δ 139.9, 130.1, 129.5, 128.9, 81.6, 71.1, 24.5; MS (EI) *m*/*z* = 150 (M^+^), 115 (100%), 89, 63; anal. C 71.76, H 4.71%, calcd for C_9_H_7_Cl, C 71.78, H 4.69%.

#### 1-Chloro-3-(prop-2-yn-1-yl)benzene (**26**):

yellow oil (151 mg, quantitative yield); IR (neat) 2154, 1491, 1212, 1160, 990, 906, 730, 652, 593, 489 cm^–1^; ^1^H NMR (CDCl_3_, 400 MHz) δ 7.38–7.36 (m, 1H), 7.25–7.22 (m, 3H), 3.61–3.58 (m, 2H), 2.22 (t, *J* = 2.7 Hz, 1H); ^13^C NMR (CDCl_3_, 100 MHz) δ 138.2, 134.5, 129.9, 128.7, 127.1, 126.4, 81.1, 71.2, 24.6; MS (EI) *m*/*z* = 150 (M^+^), 115 (100%), 89, 63; anal. C 71.80, H 4.72%, calcd for C_9_H_7_Cl, C 71.78, H 4.69%.

#### 1-Methoxy-3-(prop-2-yn-1-yl)benzene (**27**):

yellow oil (135 mg, 92% yield); IR (neat) 3292, 2958, 2835, 1600, 1585, 1488, 1454, 1212, 1159, 996, 737, 638, 489 cm^–1^; NMR spectra were in accordance with data reported in the literature:^27^^1^H NMR (CDCl_3_, 400 MHz) δ 7.30–7.22 (m, 1H), 6.98–6.90 (m, 2H), 6.83–6.76 (m, 1H), 3.82 (s, 3H), 3.62–3.58 (m, 2H), 2.20 (t, *J* = 2.8 Hz, 1H); ^13^C NMR (CDCl_3_, 100 MHz) δ 159.9, 137.8, 129.7, 120.3, 113.7, 112.3, 81.9, 70.7, 55.3, 24.9; MS (EI) *m*/*z* = 146 (M^+^, 100%), 131, 115, 103, 89, 63; anal. C 82.13, H. 6.94%, calcd for C_10_H_10_O, C 82.16, H 6.90%.

### Synthesis of Compounds **2**–**7**

Compounds **2**–**7** were synthesized according to the methodology developed by Tomas-Mendivil et al.,^29^ except for the products’ purification, which was performed through silica gel column chromatography (100% *n*-hexane).

#### 2-(3-(*p*-Tolyl)-prop-1-yn-1-yl)furan (**2**):

yellow oil (66 mg, 40% yield); IR (neat) 3032, 2921, 2216, 1513, 1486, 983, 900, 793, 738, 592, 475 cm^–1^; ^1^H NMR (400 MHz, CDCl_3_) δ 7.35 (m, 1H), 7.27 (d, *J* = 7.9 Hz, 2H), 7.15 (d, *J* = 7.7 Hz, 2H), 6.52 (d, *J* = 2.6 Hz, 1H), 6.39–6.35 (m, 1H), 3.81 (s, 2H), 2.34 (s, 3H); ^13^C NMR (CDCl_3_, 100 MHz) δ 142.7, 137.2, 136.2, 132.7, 129.1, 127.7, 114.0, 110.5, 92.1, 72.5, 25.2, 20.8; MS (EI) *m*/*z* = 196 (M^+^, 100%), 181, 167, 152, 128, 115, 91, 51; anal. C 85.65, H 6.19%, calcd for C_14_H_12_O, C 85.68, H 6.16%.

#### 2-(3-(4-Bromophenyl)-prop-1-yn-1-yl)furan (**3**):

yellow oil (107 mg, 49% yield); IR (neat) 3033, 2925, 2218, 1515, 1488, 986, 906, 796, 740, 596, 477 cm^–1^; ^1^H NMR (500 MHz, CDCl_3_) δ 7.49 (t, *J* = 2.0 Hz, 1H), 7.48 (d, *J* = 2.0 Hz, 1H), 7.39–7.38 (m, 1H), 7.29–7.27 (m, 2H), 6.56 (d, *J* = 3.2 Hz, 1H), 6.40 (dd, *J* = 3.3, 1.9 Hz, 1H), 3.83 (s, 2H); ^13^C NMR (CDCl_3_, 100 MHz) δ 143.1, 138.2, 137.1, 135.0, 130.5, 129.7, 129.7, 123.2, 114.5, 110.7, 91.2, 73.4, 25.4; MS (EI) *m*/*z* = 261.5 (M^+^), 181 (100%), 168, 152, 115 91, 51; anal. C 59.84, H 3.49%, calcd for C_13_H_9_BrO, C 59.80, H 3.47%.

#### 2-(3-(4-(Methylthio)phenyl)-prop-1-yn-1-yl)furan (**4**):

yellow solid (96 mg, 48% yield); IR (neat) 3035, 2253, 1492, 1093, 985, 904, 727, 649, 592 cm^–1^; ^1^H NMR (500 MHz, CDCl_3_) δ 7.38 (dd, *J* = 1.8, 0.7 Hz, 1H), 7.35–7.31 (m, 2H), 7.28–7.27 (m, 1H), 7.27–7.25 (m, 1H), 6.55 (d, *J* = 3.4 Hz, 1H), 6.39 (dd, *J* = 3.4, 1.9 Hz, 1H), 3.83 (s, 2H), 2.50 (s, 3H); ^13^C NMR (CDCl_3_, 100 MHz) δ 143.0, 137.3, 136.8, 132.9, 128.5, 128.4, 127.2, 127.1, 114.3, 110.7, 91.8, 73.0, 25.3, 16.2; MS (EI) *m*/*z* = 228 (M^+^), 181 (100%), 152, 126, 91, 51; anal. C 73.66, H 5.33, S 14.06%, calcd for C_14_H_12_OS, C 73.65, H 5.30, S 14.04%.

#### 2-(3-(4-Chlorophenyl)-prop-1-yn-1-yl)furan (**5**):

yellow oil (82 mg, 44% yield); IR (neat) 3034, 2925, 2218, 1596, 1574, 1431, 1077, 986, 904, 775, 741, 592, 432 cm^–1^; ^1^H NMR (400 MHz, CDCl3) δ 7.39–7.37 (m, 1H), 7.37 (dd, *J* = 1.9, 0.7 Hz, 1H), 7.28 (s, 1H), 7.27–7.25 (m, 2H), 6.55 (d, *J* = 3.4 Hz, 1H), 6.38 (dd, *J* = 3.4, 1.9 Hz, 1H), 3.82 (s, 2H); ^13^C NMR (CDCl_3_, 100 MHz) δ 143.4, 138.1, 137.3, 134.7, 130.1, 128.4, 127.3, 126.4, 114.9, 110.9, 91.2, 73.7, 25.8; MS (EI) *m*/*z* = 216 (M^+^), 181 (100%), 152, 127, 92, 51; anal. C 72.10, H 4.21%, calcd for C_13_H_9_ClO, C 72.07, H 4.19%.

#### 2-(3-(3-Chlorophenyl)-prop-1-yn-1-yl)furan (**6**):

yellow oil (48 mg, 37% yield); IR (neat) 3004, 1598, 1577, 1474, 1432, 1264, 1078, 895, 855, 777, 703, 643, 436 cm^–1^; ^1^H NMR (400 MHz, CDCl_3_) δ 7.39–7.38 (m, 1H), 7.37 (dd, *J* = 1.9, 0.7 Hz, 1H), 7.27 (t, *J* = 1.4 Hz, 1H), 7.27–7.25 (m, 2H), 6.55 (d, *J* = 3.4 Hz, 1H), 6.38 (dd, *J* = 3.4, 1.9 Hz, 1H), 3.83 (s, 2H); ^13^C NMR (CDCl_3_, 100 MHz) δ 143.4, 138.1, 137.3, 134.7, 130.1, 128.4, 127.3, 126.4, 114.9, 111.1, 91.2, 73.7, 25.8; MS (EI) *m*/*z* = 216 (M^+^, 100%), 152, 127, 92, 51; anal. C 72.10, H 4.21%, calcd for C_13_H_9_ClO, C 72.07, H 4.19%.

#### 2-(3-(3-Methoxyphenyl)-prop-1-yn-1-yl)furan (**7**):

yellow oil (134 mg, 63% yield); IR (neat) 2938, 2835, 1600, 1585, 1487, 1464, 1453, 1257, 1152, 1048, 984, 900, 775, 737, 592, 432 cm^–1^; ^1^H NMR (400 MHz, CDCl_3_) δ 7.35 (dd, *J* = 1.9, 0.7 Hz, 1H), 7.25 (s, 1H), 6.99–6.97 (m, 1H), 6.97–6.94 (m, 1H), 6.80 (dd, *J* = 8.0, 2.3 Hz, 1H), 6.53 (d, *J* = 3.4 Hz, 1H), 6.37 (dd, *J* = 3.4, 1.9 Hz, 1H), 3.83 (s, 2H), 3.82 (s, 3H); ^13^C NMR (CDCl_3_, 100 MHz) δ 160.1, 143.2, 137.7, 137.5, 129.8, 120.6, 114.5, 114.0, 112.5, 110.9, 92.0, 73.2, 55.5, 26.1; MS (EI) *m*/*z* = 212 (M^+^, 100%), 181, 169, 152, 127, 115, 51; anal. C 79.20, H 5.75%, calcd for C_14_H_12_O_2_, C 79.23, H 5.70%.

### *Carlina acaulis* Essential Oil Isolation and Chemical Characterization

Dry roots of *C. acaulis* were purchased from A. Minardi & Figli (Bagnacavallo, Ravenna, Italy). The EO was obtained through hydrodistillation (HD) from the roots preventively reduced to a 1.5 mm size and following the procedure reported by Benelli et al.^45^ A 1 kg amount of roots was soaked for 16 h with 7 L of distilled water in a 10 L round-bottom flask. The HD process was conducted accordingly employing the distillation system previously reported,^45^ and the EO was obtained in 0.97% yield (w/w) (yellowish color, density of 1.063 g mL^–1^, and refractive index of 1.584). The EO was chemically characterized by GC-MS analysis and instrumental and analytical conditions, together with a chromatogram study following those previously published.^45^

### Carlina Oxide Isolation

Part of the obtained EO (1.403 g) was subjected to silica gel (70 g) column chromatography (70–230 mesh, 60 Å, Merck) using *n*-hexane (Merck, Italy) as eluent. Carlina oxide (**1**) (1.306 g) was isolated and then characterized by NMR and mass spectrometry analyses. The chemical identification obtained was in accordance with that already reported.^31^

### Insecticidal Assays

#### Houseflies

The used houseflies, *M. domestica* (females, 3–5 days old), were obtained from an established laboratory colony (Crop Research Institute, Czech Republic, >20 generations). Houseflies were reared as detailed by Pavela^46^ and were maintained at 25 ± 1 °C, 50–70% RH, and 16:8 (L:D). Larvae were reared in a mixture of sterilized bran, milk powder, and water; adults were provided with ad libitum access to water and to milk powder.

#### Mosquitoes

*Cx. quinquefasciatus* 3rd instar larvae were obtained from an established laboratory colony (Crop Research Institute, Czech Republic, >20 generations) as well. The larvae were fed on dog biscuits and yeast powder in a 3:1 ratio. Rearing conditions were 25 ± 2 °C, 70 ± 5% RH, and 16:8 (L:D) h.

#### Topical Bioassays on Houseflies

Acute topical toxicity of *C. acaulis* EO, carlina oxide, and its analogues on *M*. *domestica* adult females was evaluated according to Pavela.^46^ Fly females were anaesthetized using CO_2_. The products were diluted in acetone (p.a. purity, Sigma-Aldrich, Czech Republic), employing a concentration series to obtain the following doses upon application of 1 μL onto the housefly pronotum: 1, 3, 5, 8, 12, 15, 18, 20, 25, 30, 35, and 40 μg fly^–1^. To calculate the lethal doses alone, 5 or 6 doses were selected, which caused mortality in the range of 20–90%. The doses were applied using a microelectric applicator; the control flies were treated only with the solvent used for dilutions. Groups of 20 adults, replicated 4 times, were tested for each dose. After evaporation of acetone (approximately after 3–5 min), the flies were transferred to air-permeable plastic boxes (10 × 15 × 8 cm) containing food in the form of a 20% sugar solution (w:v). The experiment was performed in an air-conditioned room at 25 ± 1 °C, 70 ± 3% RH, and 16:8 h (L:D). The entire experiment was repeated 4 times. Adult mortality was evaluated 24 h after treatment.

#### Acute Toxicity on Mosquito Larvae

Acute toxicity of *C. acaulis* EO, carlina oxide, and its analogues diluted in DMSO (Sigma-Aldrich, Czech Republic) for *Cx. quinquefasciatus* larvae was evaluated following the World Health Organization (WHO)^47^ procedure with minor modifications by Benelli et al.^31^ Experimental treatment was prepared as follows: 1 mL of serial dilution was dissolved using DMSO in 224 mL of distilled water using a 500 mL glass bowl and shaken to produce a homogeneous test solution. The tested concentrations were 0.5, 0.8, 1.0, 1.2, 1.4, 1.6, 1.8, 2.0, 2.2, 2.4, and 2.6 μg mL^–1^, while each concentration was replicated 4 times on groups of 25 larvae/beaker each. Distilled water containing the same amount of DMSO as that used to dissolve the compounds was used as the negative control. *Cx. quinquefasciatus* larvae were transferred into water in the bowl containing the prepared test solution (25 larvae/beaker). Four duplicate trials (100 larvae per single replication) were performed for each sample concentration, while each trial included a negative control composed of distilled water with the same amount of DMSO as the test sample. The assays were put in a growth chamber [25 ± 1 °C; 16:8 (L:D)]; mortality was recorded after 24 h.

#### Sublethal Effects on Mosquito Larvae

To assess the effect of low concentrations (i.e., LC_30_) of *C. acaulis* EO, carlina oxide, and its analogues on *Cx. quinquefasciatus*, 3rd instar larvae were exposed to each product for 24 h (the used concentrations are presented in Table 3). Subsequently, the surviving larvae were moved to clean water and nourished with a standard diet. The application methods are detailed in the paragraph dedicated to larvicidal tests. Larval and pupal mortality and emerged adults were evaluated, and 4 replicates were completed for each product tested. Again, the treated insects were positioned in a growth chamber (25 ± 1 °C; 16:9 (L:D)).

#### Mosquito Larval Mortality over Time When Exposed to LC_90_

The increase of *Cx. quinquefasciatus* mortality rate in time upon application of LC_90_ concentrations was evaluated as follows. The EO, carlina oxide, or the analogues were mixed in water using the same method as reported for acute toxicity tests. The tested concentrations are given in Table 4. Mortality was assessed at different time intervals, i.e., 4, 8, 12, 16, 20, and 24 h, from introducing the larvae in water contaminated with respective substances. Larvae not reacting to mechanical stimuli were considered dead. At the time of each mortality check, dead larvae were removed using a brush; 4 replicates (25 larvae/each) were performed for each product tested. Mosquitoes were placed in a growth chamber (25 ± 1 °C; 16:8 (L:D)).

### Cytotoxicity Assays

#### Cell Lines

Immortalized human keratinocytes cell line (HaCaT), provided by IFO (Istituti Fisioterapici Ospitalieri, Rome, Italy), was cultured in DMEM enriched with 10% fetal bovine serum (FBS), 100 IU mL^–1^ penicillin/streptomycin, and 2 mM l-glutamine and kept at 37 °C with 5% CO_2_ and 95% humidity.

#### Cell Viability Assay

Cells were seeded at a density of 2 × 10^3^/well in a 96-well plate with a final volume of 100 μL. After overnight incubation, cells were treated with different concentrations of carlina oxide analogues, *C. acaulis* EO, and carlina oxide (up to 100 μg mL^–1^) for 3 days, and then cytotoxicity was evaluated by adding MTT. After 3 h, the salt crystals were solubilized in 100 μL/well of DMSO. An ELISA reader microliter plate (BioTek Instruments, Winooski, VT, USA) was employed for the measurement of the absorbance of samples at 570 nm against a control.

### Statistical Analysis

In insecticidal tests, mortality was corrected through the Abbott’s formula.^48^ LD_50(90)_ and LC_50(90)_ were estimated by probit analysis.^49^ Data in % were transformed using the arcsine square root transformation before being analyzed by ANOVA followed by Tukey’s HSD test (*p* ≤ 0.05).

Cell cytotoxicity data represent the mean with standard deviation (SD) of at least three independent experiments. Significant differences were assessed by one-way ANOVA, followed by Tukey’s HSD multiple comparisons test (*p* < 0.05). IC_50_ was calculated using GraphPad Prism software.

## Supporting Information Available

NMR spectra of compounds **2**–**7**, **15**–**20**, and **22**–**27** and GC-MS chromatogram of *Carlina acaulis* EO (PDF)
