# Supplementary material for: Synthesis of Carlina Oxide Analogues and Evaluation of Their Insecticidal Efficacy and Cytotoxicity
Source: J Nat Prod. 2023 May 12;86(5):1307–16. doi: 10.1021/acs.jnatprod.3c00137 (PMC10226105; doi:10.1021/acs.jnatprod.3c00137)

Supporting information for

Synthesis of carlina oxide analogues and evaluation of their insecticidal efficacy and cytotoxicity

Eleonora Spinozzi[a,*], *Marta Ferrati*[a], *Cecilia Baldassarri*[a], *Filippo Maggi*[a], *Roman Pavela*[b,c], *Giovanni Benelli*[d], *Cristina Aguzzi*[e], *Laura Zeppa*[e], *Loredana Cappellacci*[a], *Alessandro Palmieri*[f], *Riccardo Petrelli*[a]

[a] Chemistry Interdisciplinary Project (ChIP), School of Pharmacy, University of Camerino, Via Madonna delle Carceri, 62032 Camerino, Italy; [*] E-mail address: eleonora.spinozzi@unicam.it. Tel: +39 3343348505. [b] Crop Research Institute, Drnovska 507, 161 06 Prague 6, Czech Republic. [c] Department of Plant Protection, Czech University of Life Sciences Prague, Kamycka 129, 165 00 Praha 6, Suchdol, Czech Republic. [d] Department of Agriculture, Food and Environment, University of Pisa, Via del Borghetto 80, 856124 Pisa, Italy. [e] School of Pharmacy, University of Camerino, Via Madonna delle Carceri 9/C, 62032 Camerino, Italy. [f] School of Science and Technology, Chemistry Division, University of Camerino, Via Madonna delle Carceri, 62032 Camerino, Italy.

Table of contents

S1. NMR spectra of compounds 15-20……………………………...………………………2
S2. NMR spectra of compounds 22-27……………………………...………………………8
S3. NMR spectra of compounds 2-7………………………………...……………………..14
S4. GC-MS chromatogram of *Carlina acaulis* essential oil………………………..…………..20

S1. NMR spectra of compounds 15-20

^{1}H NMR ($CDCl_3$, 400 MHz)

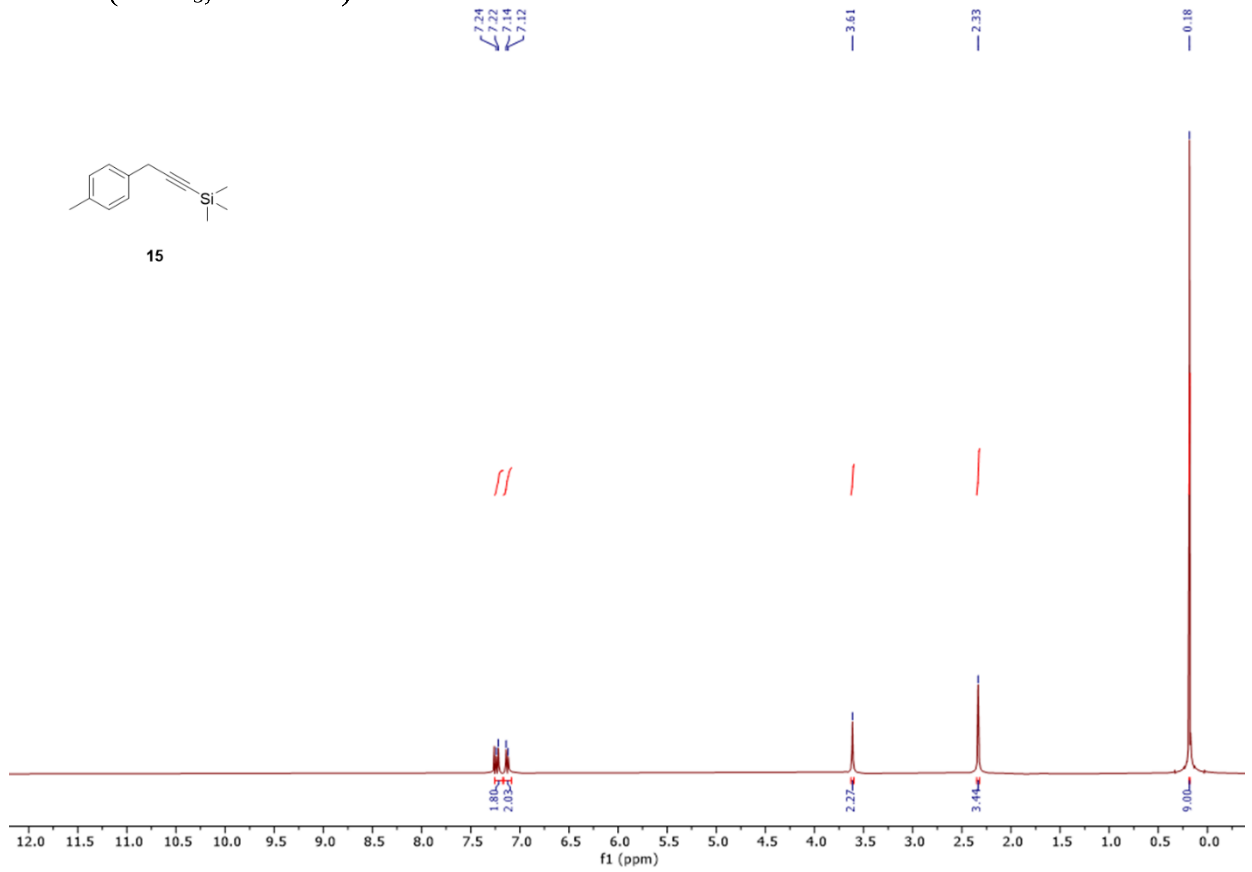

^{13}C ($CDCl_3$, 100 MHz)

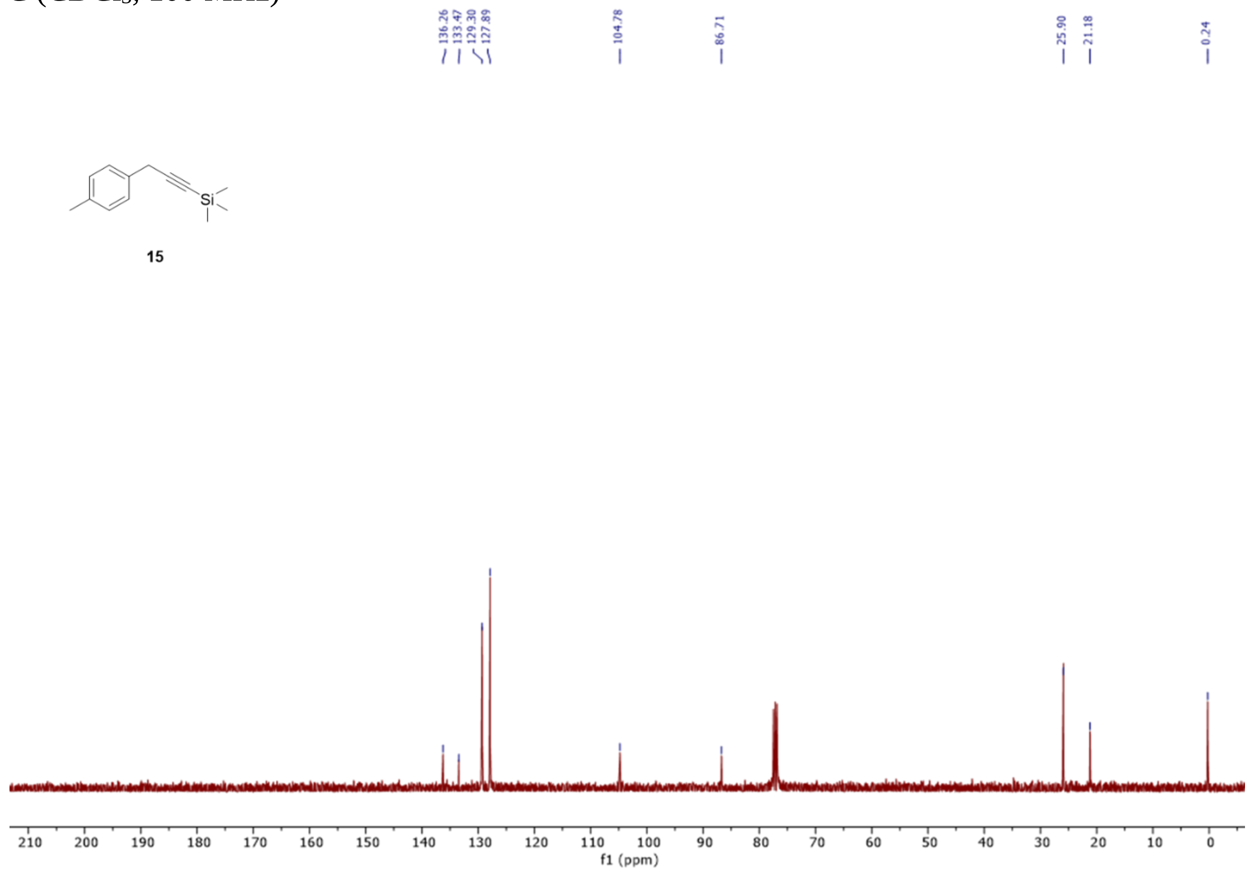

^{1}H NMR ($CDCl_3$, 500 MHz)

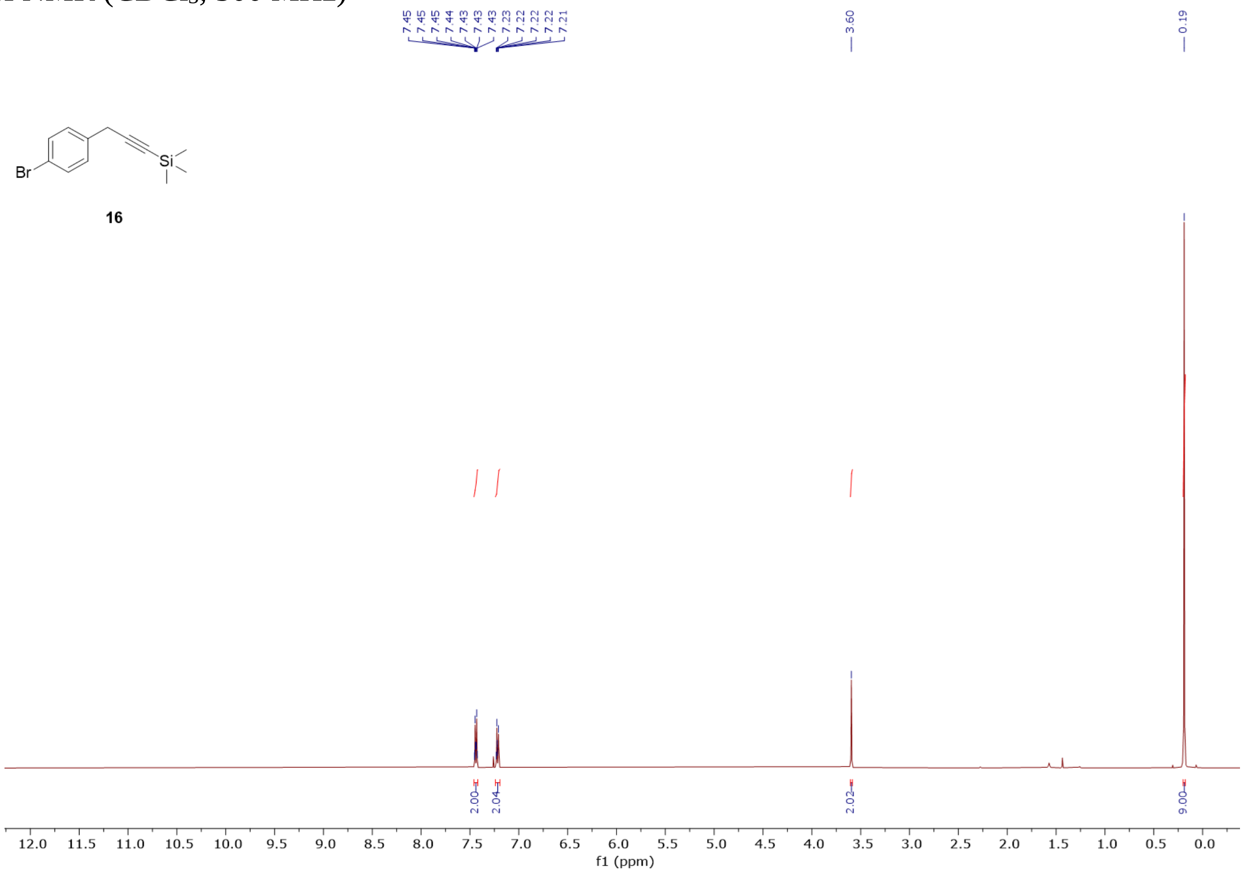

^{13}C ($CDCl_3$, 100 MHz)

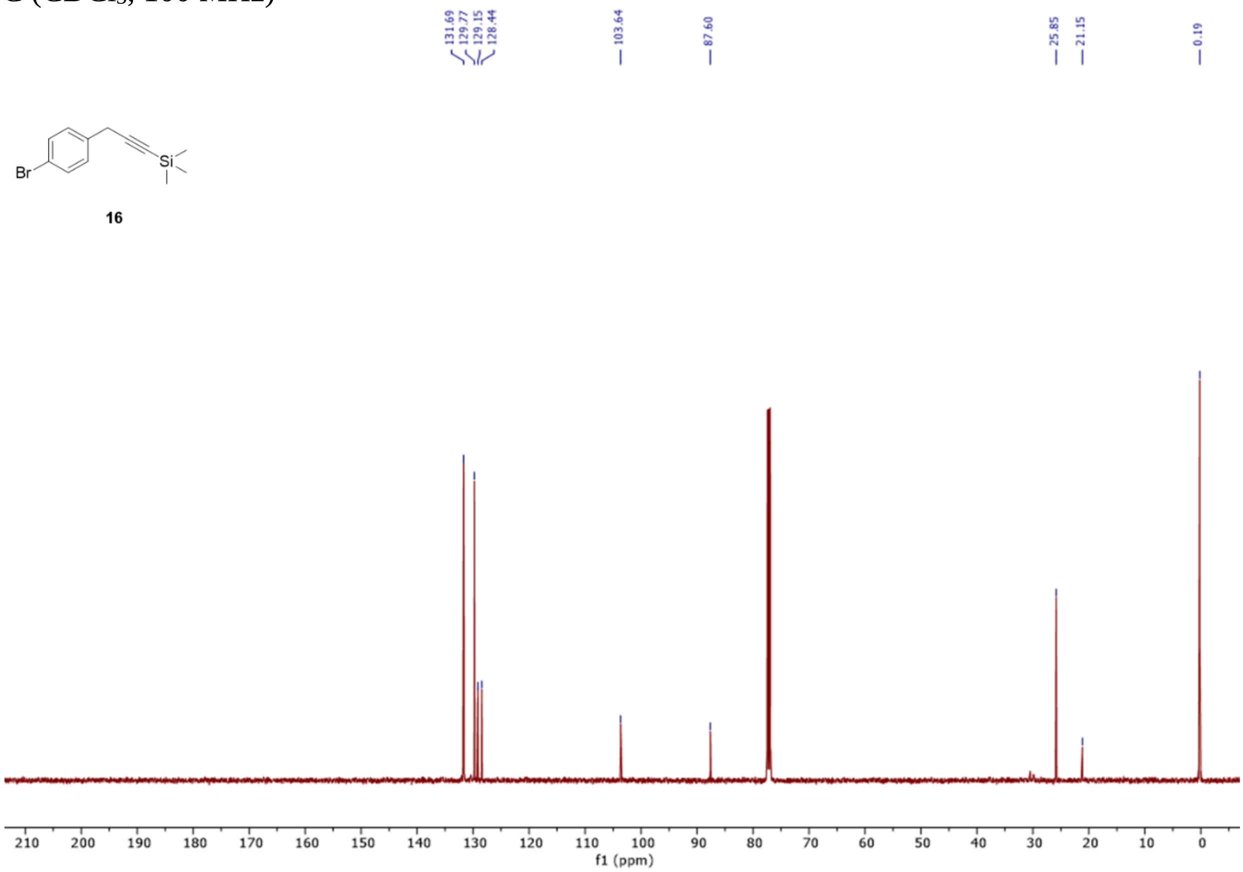

^{1}H NMR ($CDCl_3$, 500 MHz)

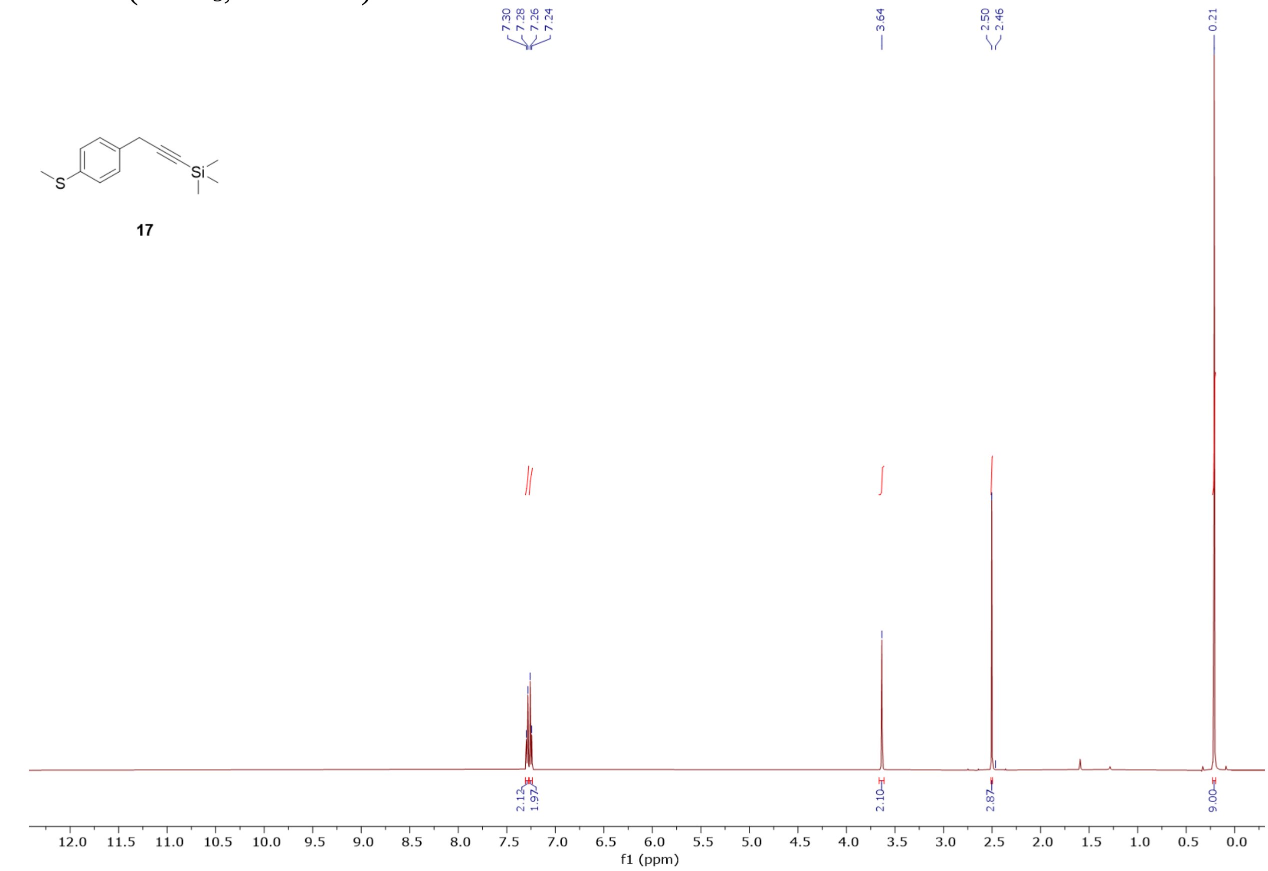

^{13}C ($CDCl_3$, 100 MHz)

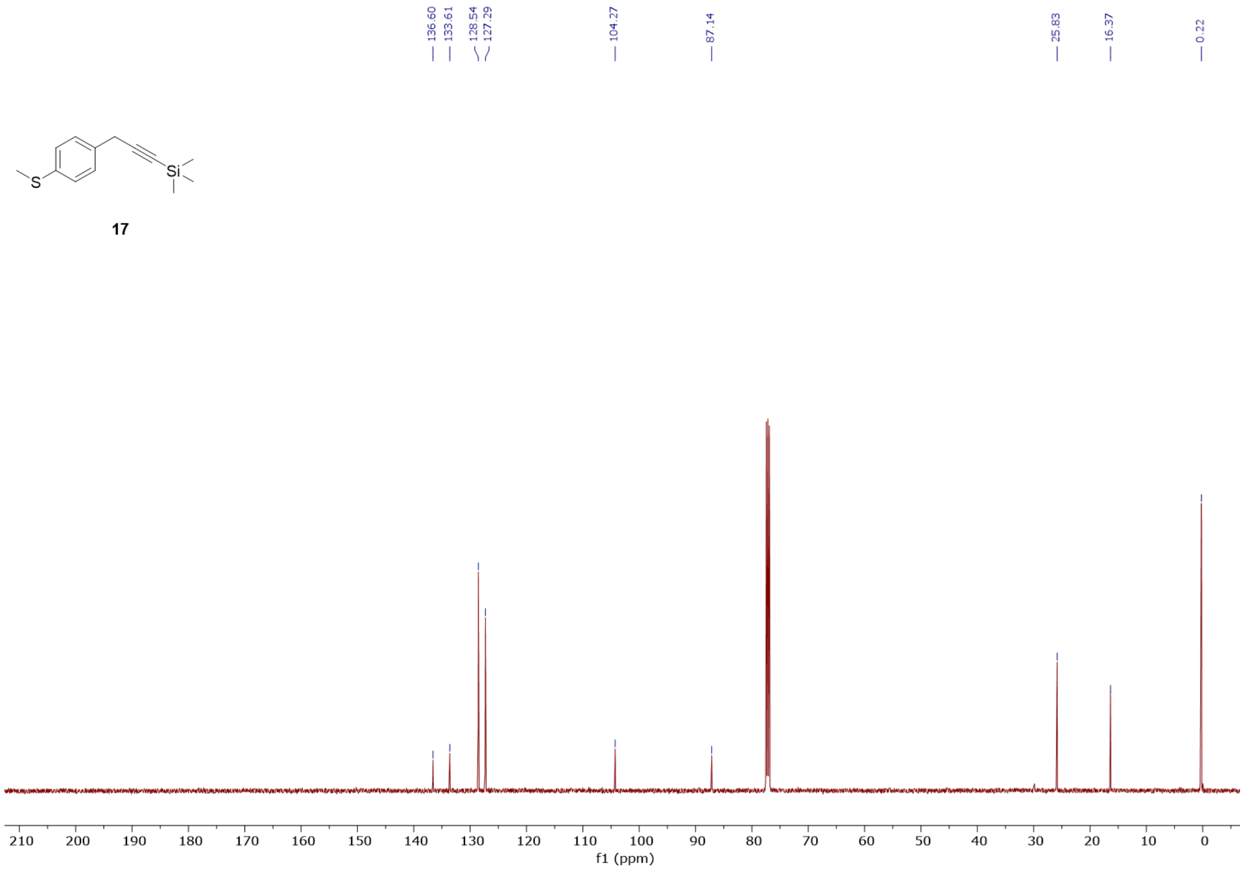

^{1}H NMR ($CDCl_3$, 500 MHz)

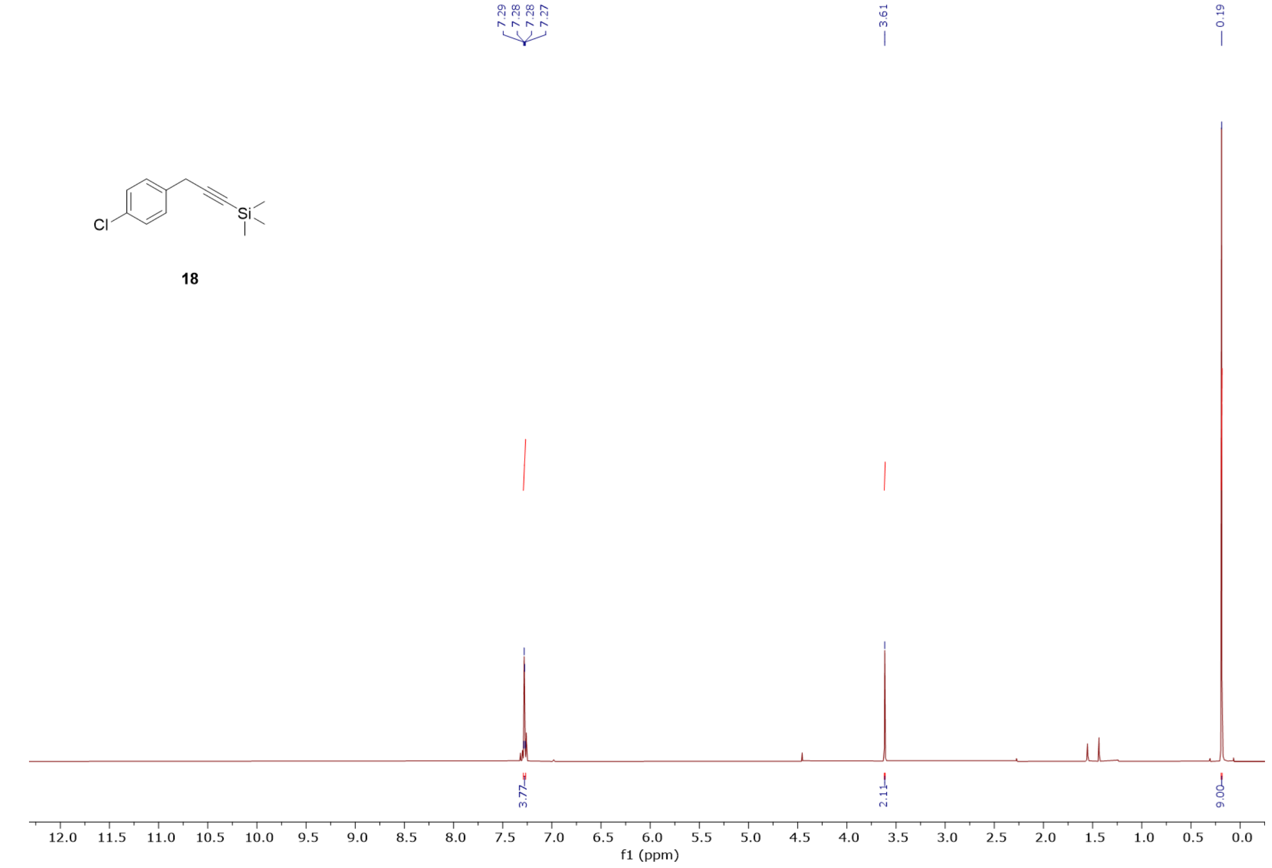

^{13}C ($CDCl_3$, 100 MHz)

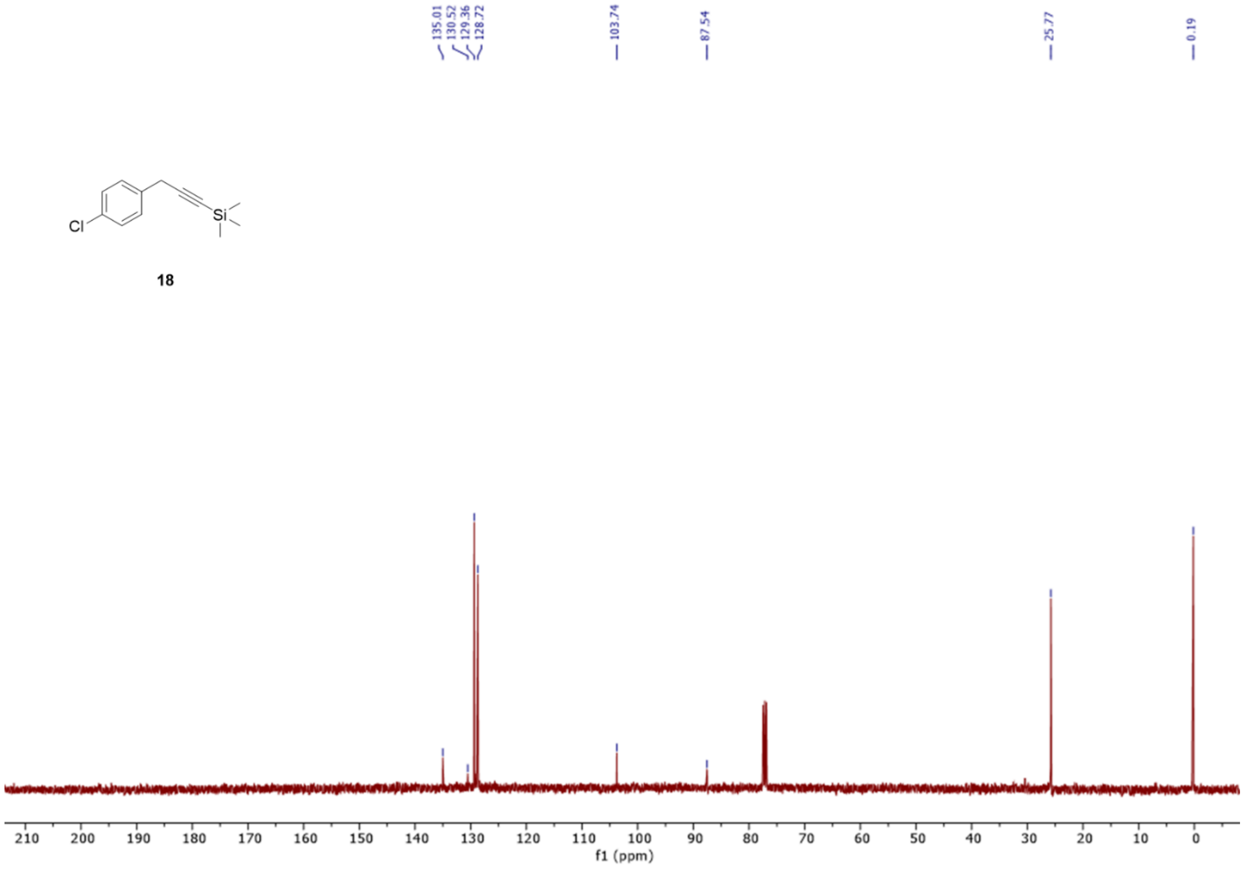

^{1}H NMR ($CDCl_3$, 400 MHz)

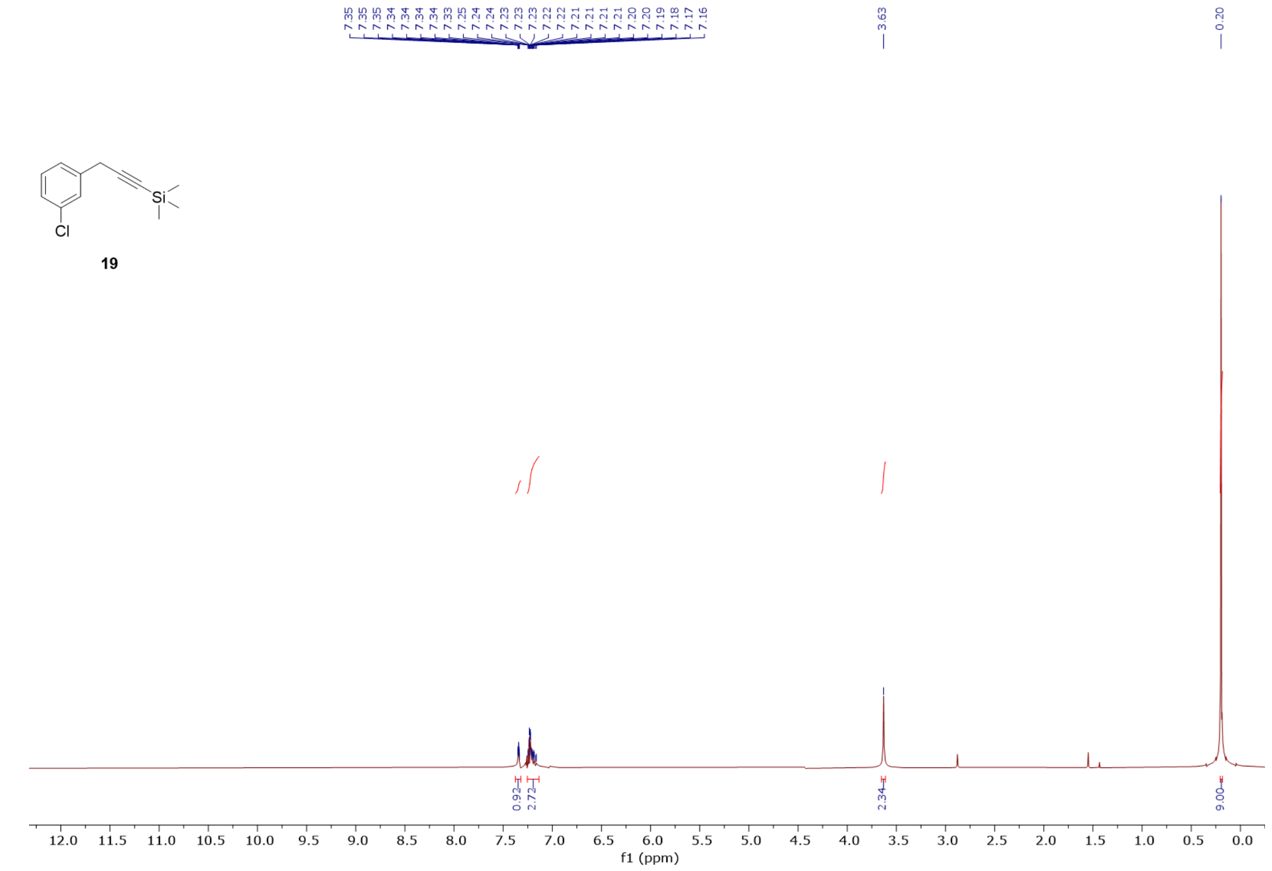

^{13}C ($CDCl_3$, 100 MHz)

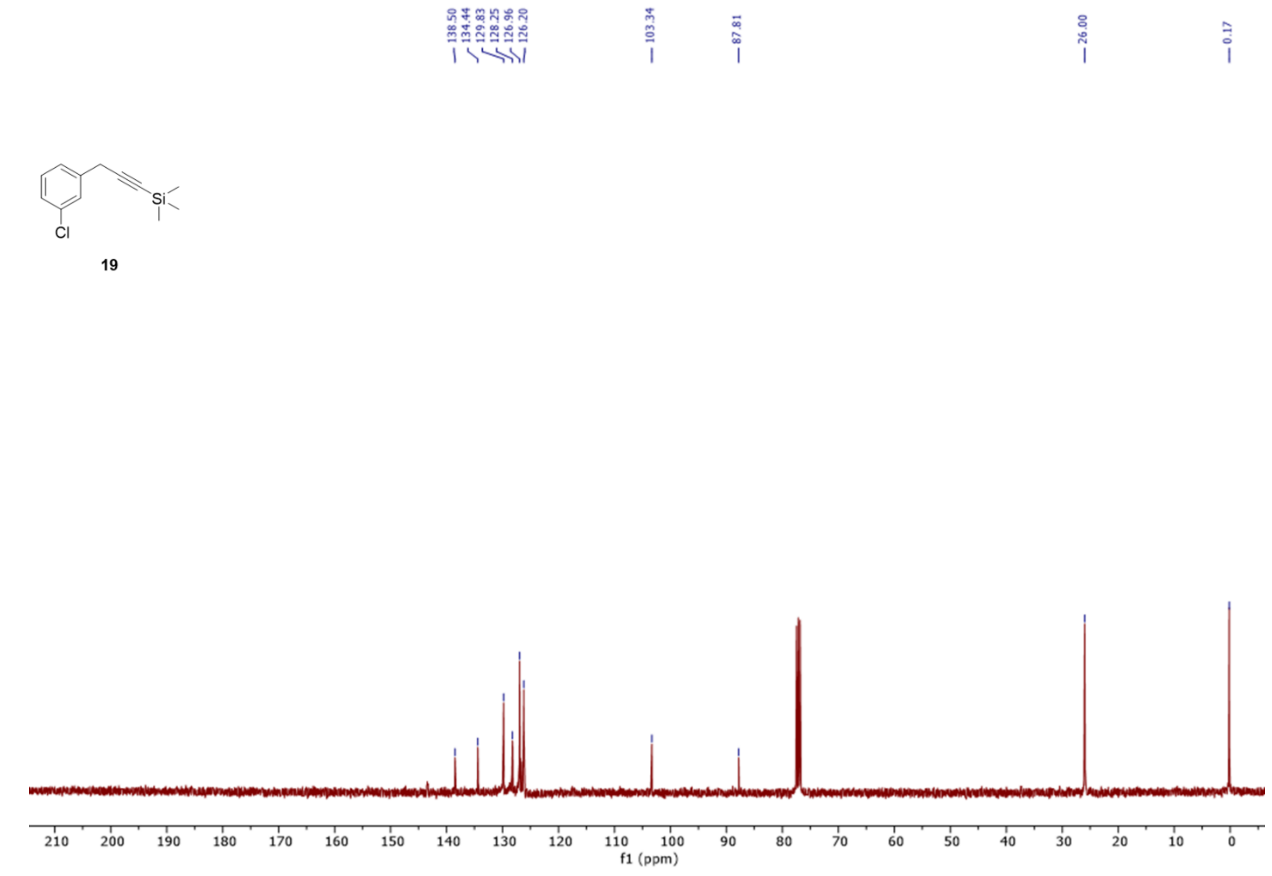

^{1}H NMR ($CDCl_3$, 400 MHz)

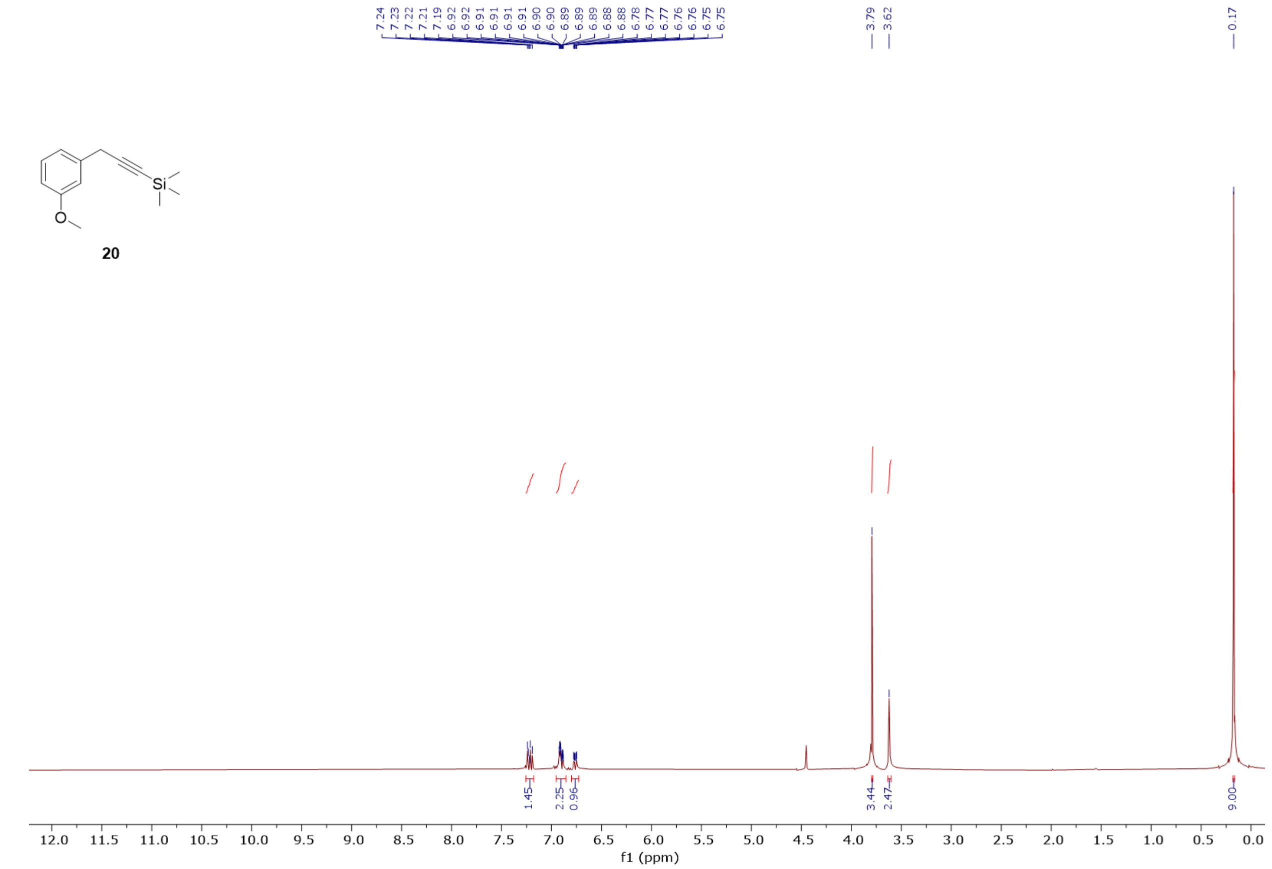

^{13}C ($CDCl_3$, 100 MHz)

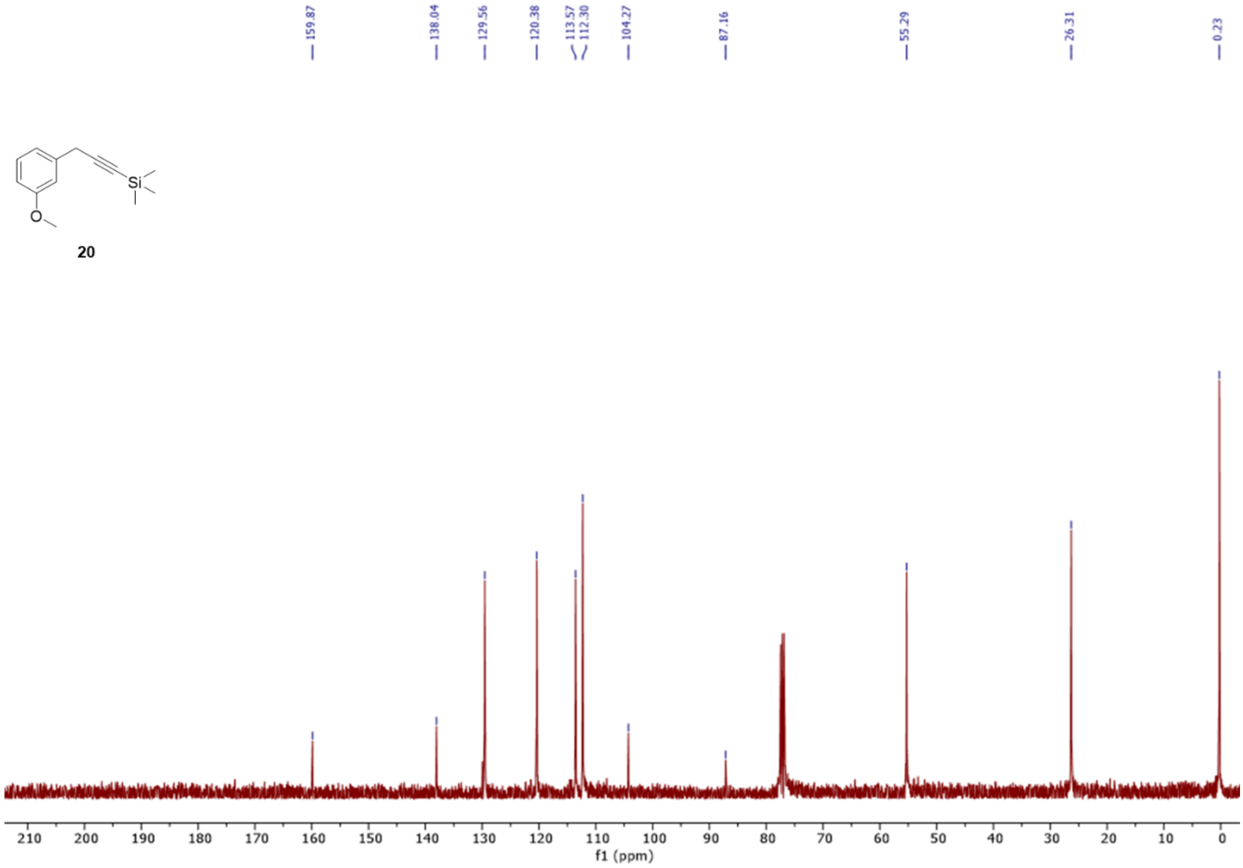

S2. NMR spectra of compounds 22-27

^{1}H NMR ($CDCl_3$, 500 MHz)

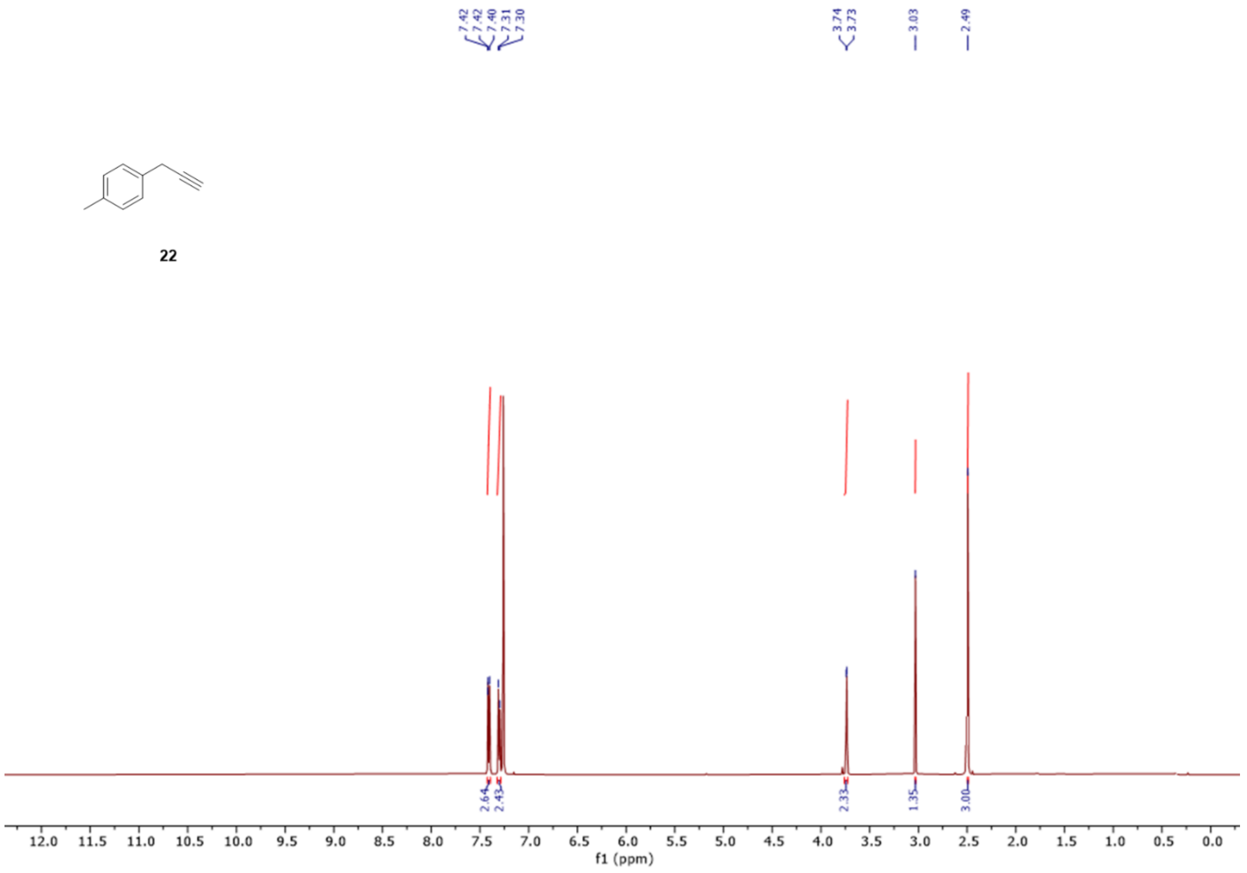

^{13}C ($CDCl_3$, 100 MHz)

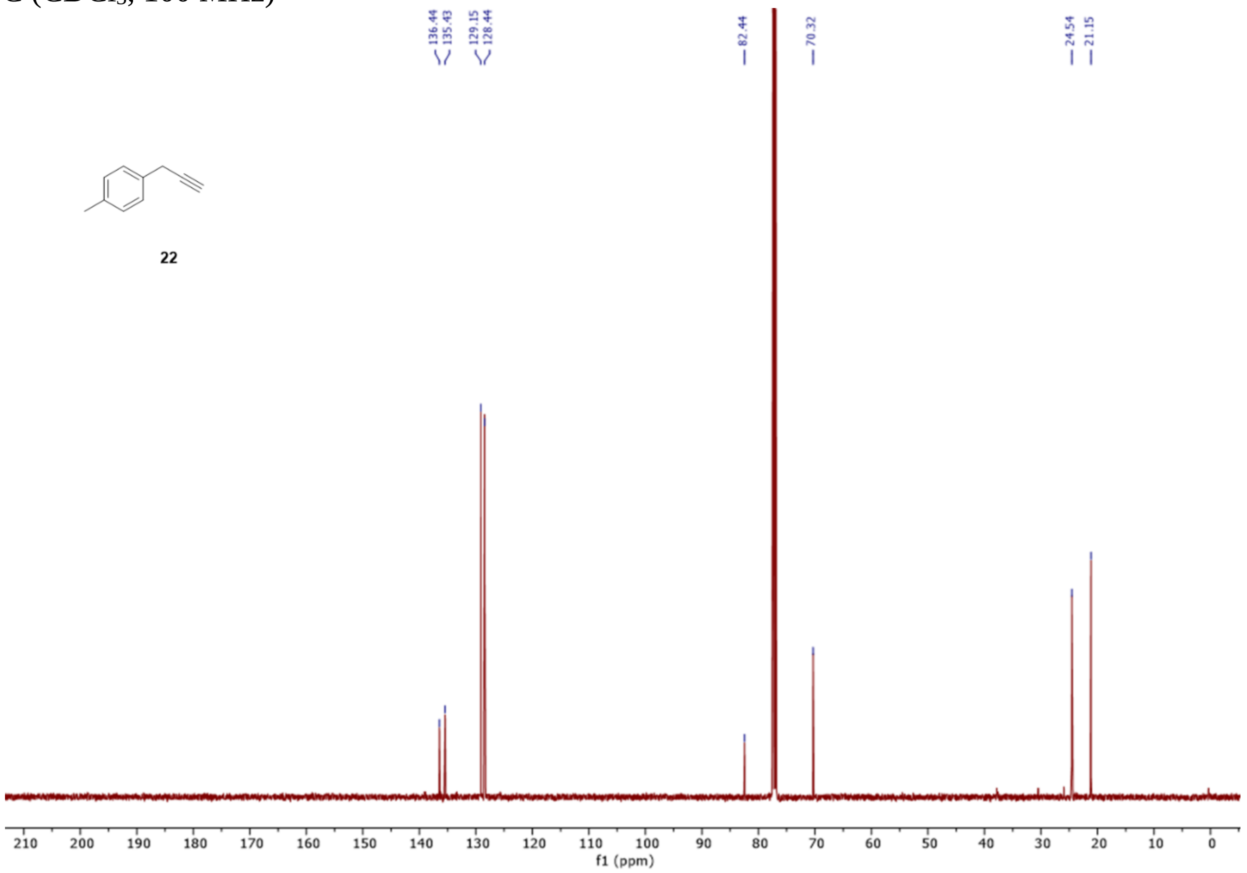

^{1}H NMR ($CDCl_3$, 500 MHz)

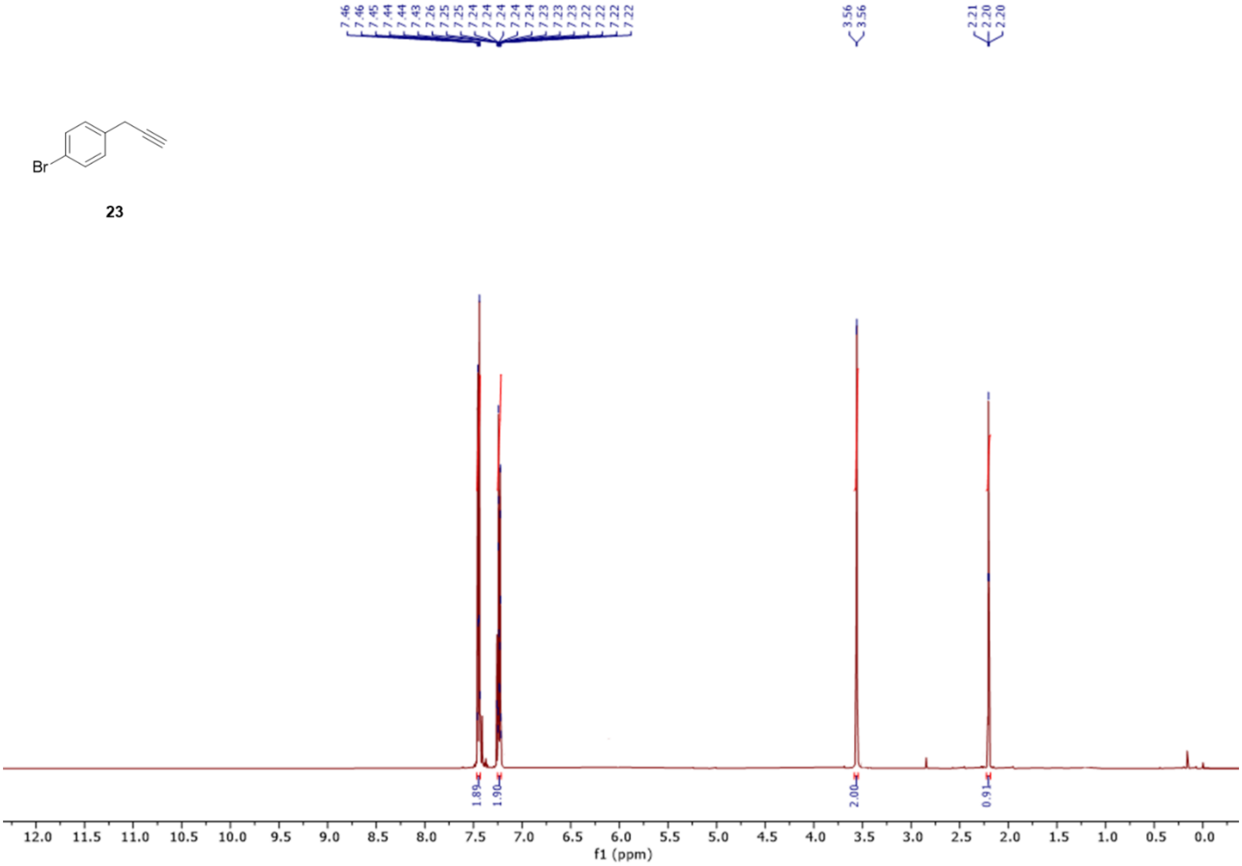

^{13}C ($CDCl_3$, 100 MHz)

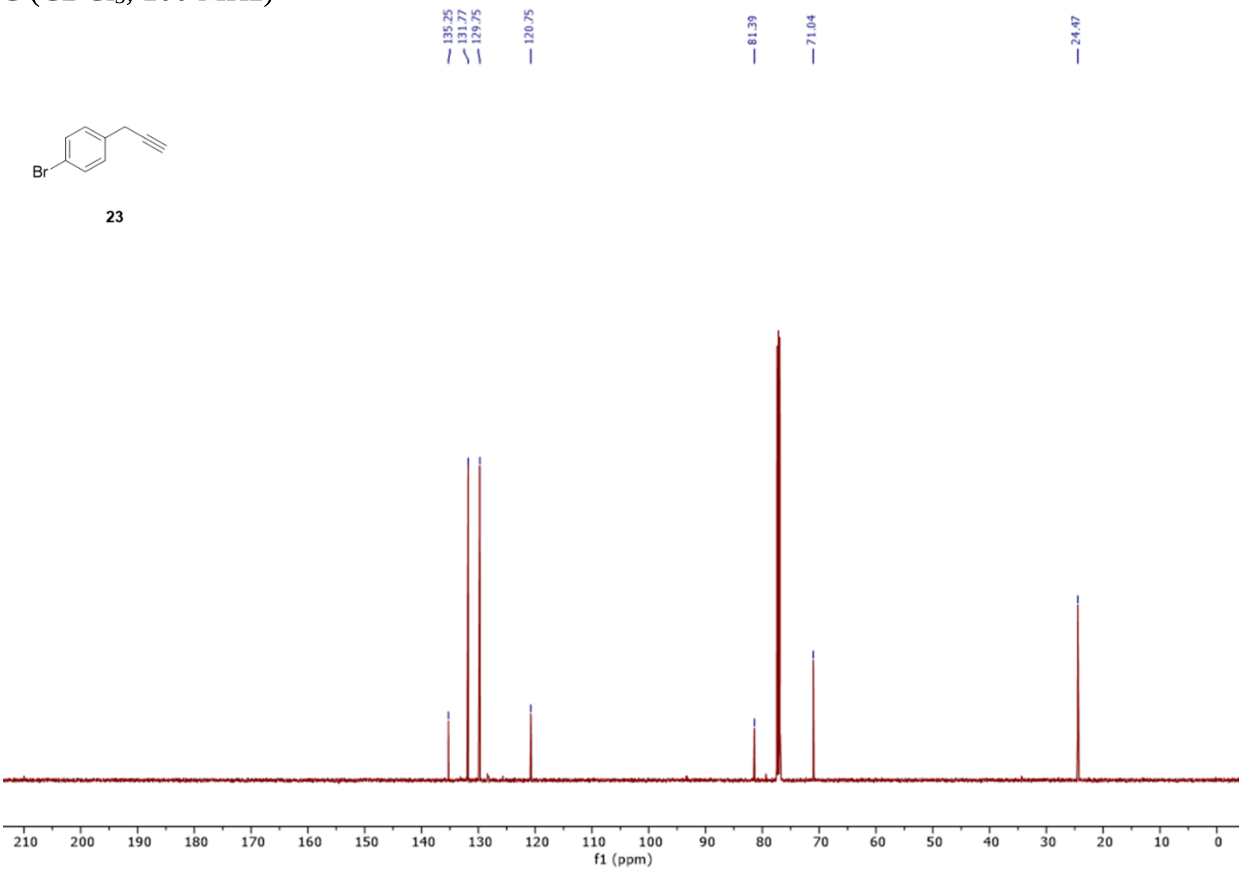

^{1}H NMR ($CDCl_3$, 500 MHz)

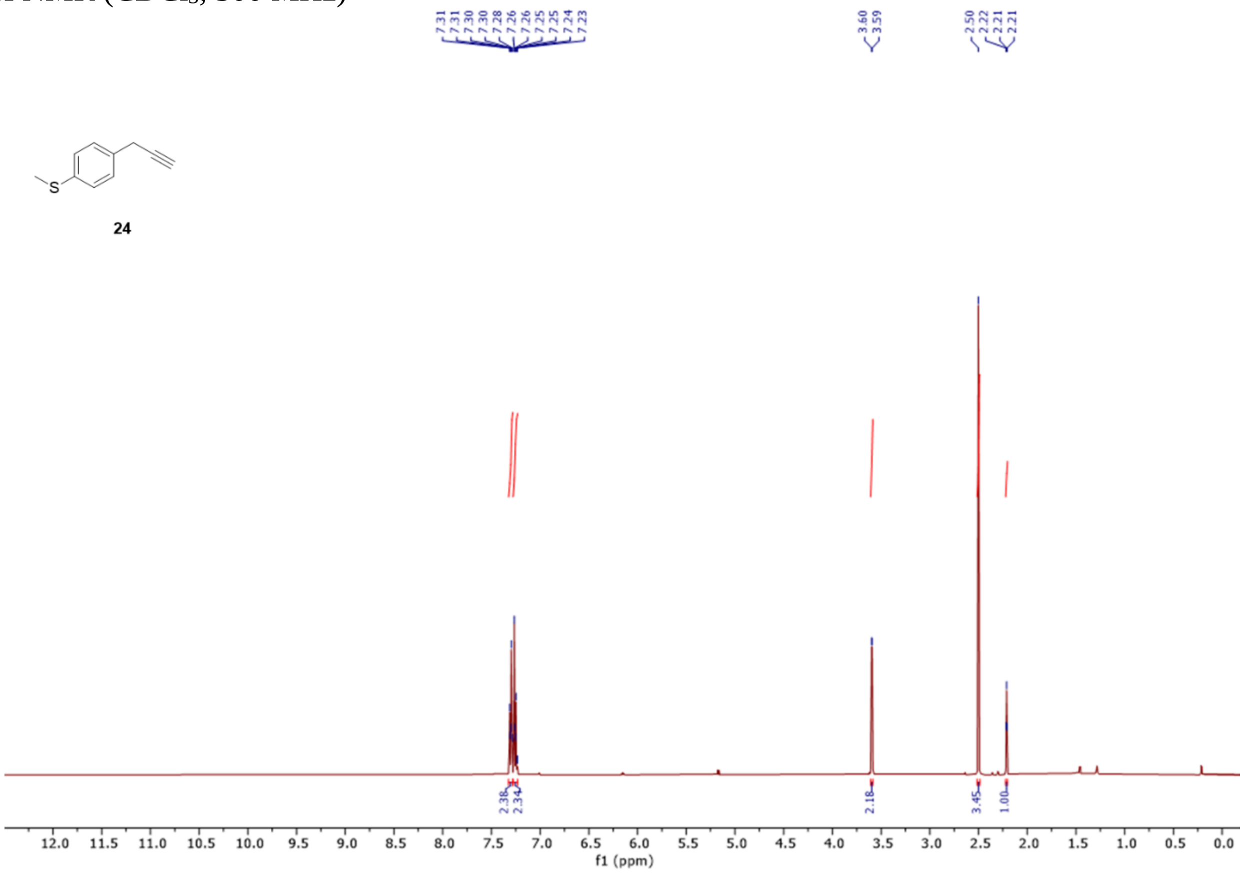

^{13}C ($CDCl_3$, 100 MHz)

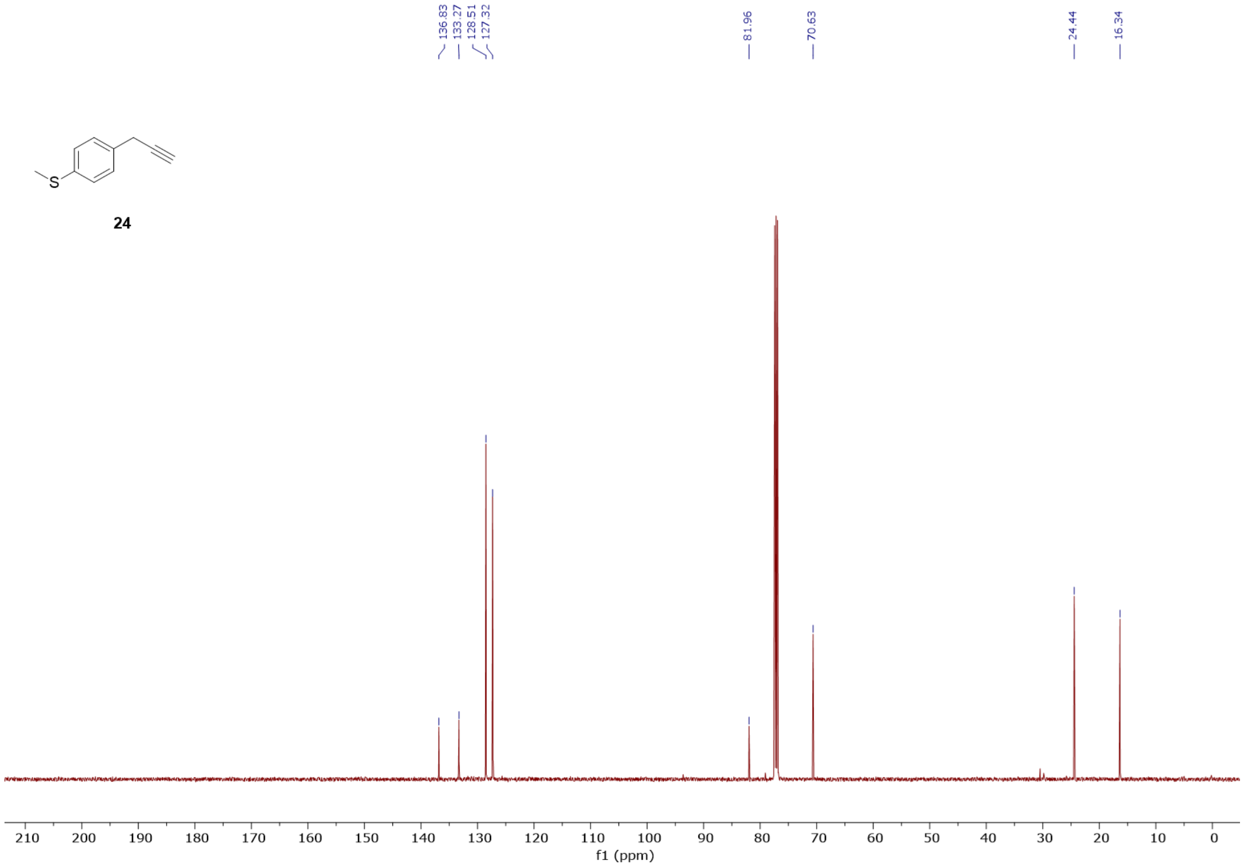

^{1}H NMR ($CDCl_3$, 500 MHz)

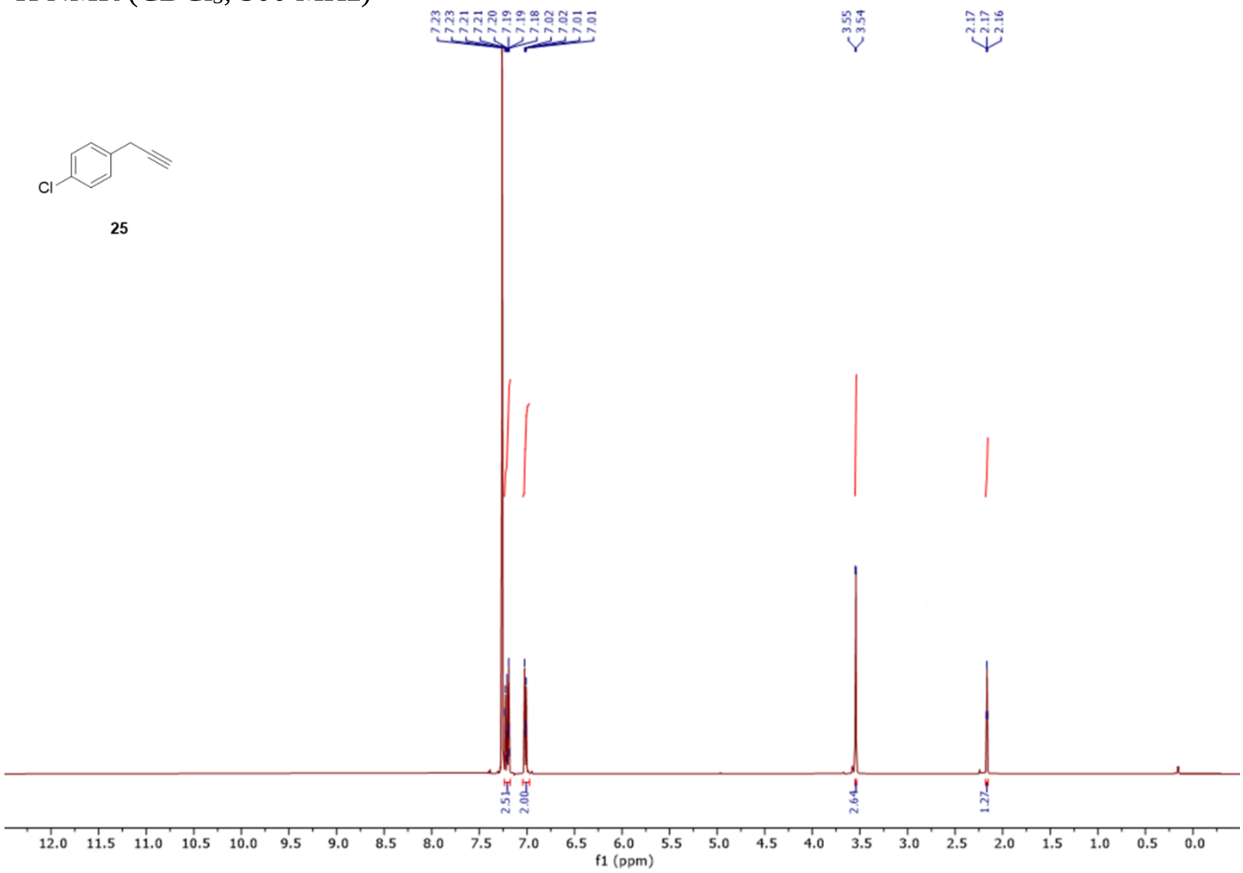

^{13}C ($CDCl_3$, 100 MHz)

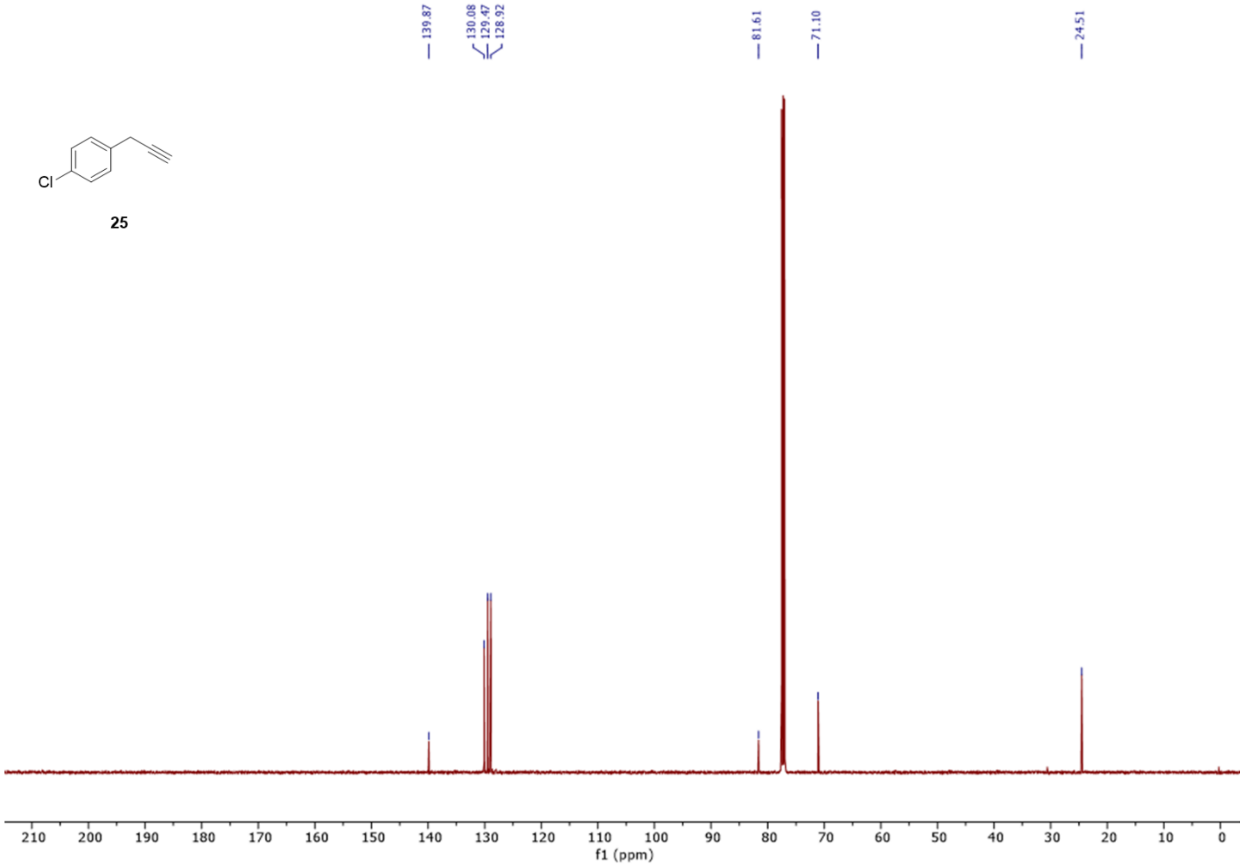

^1H NMR ($CDCl_3$, 400 MHz)

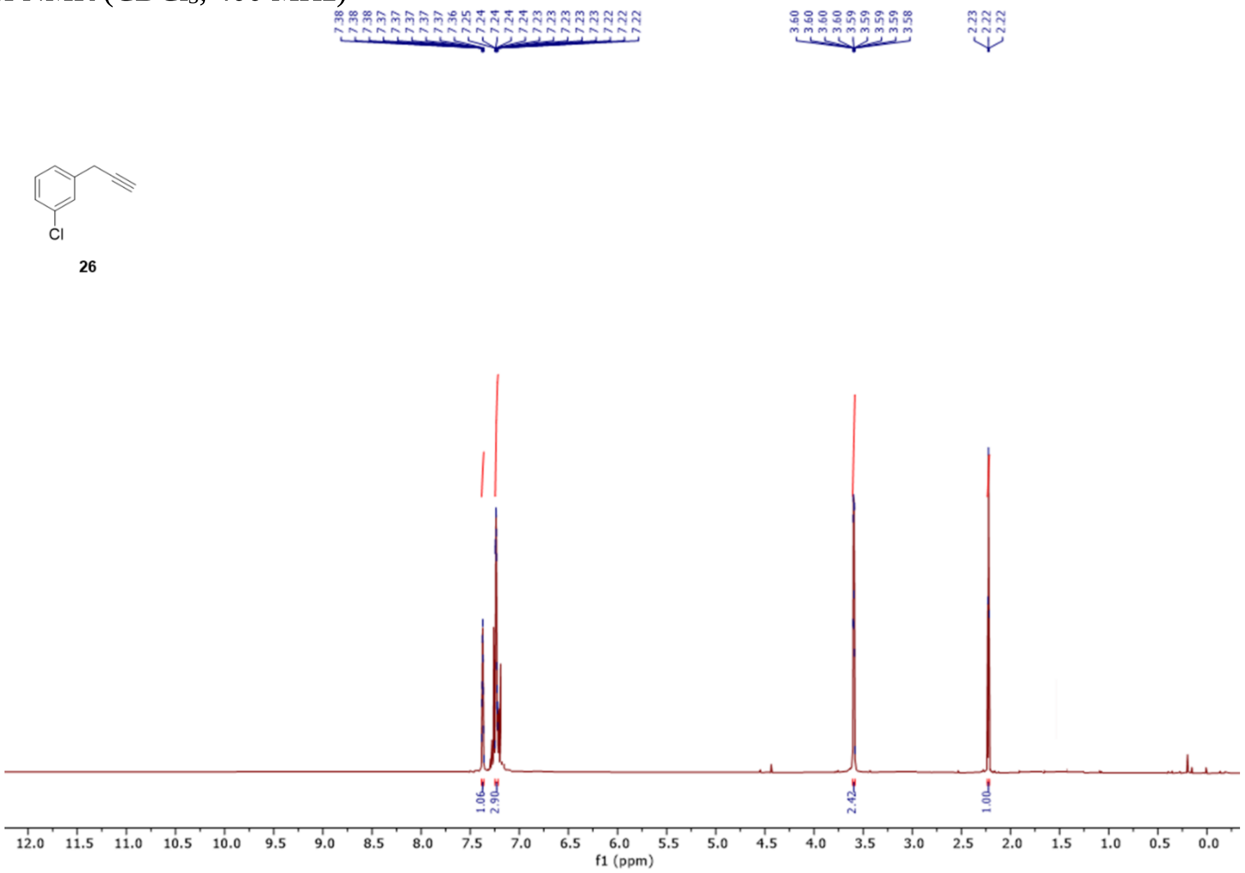

^{13}C ($CDCl_3$, 100 MHz)

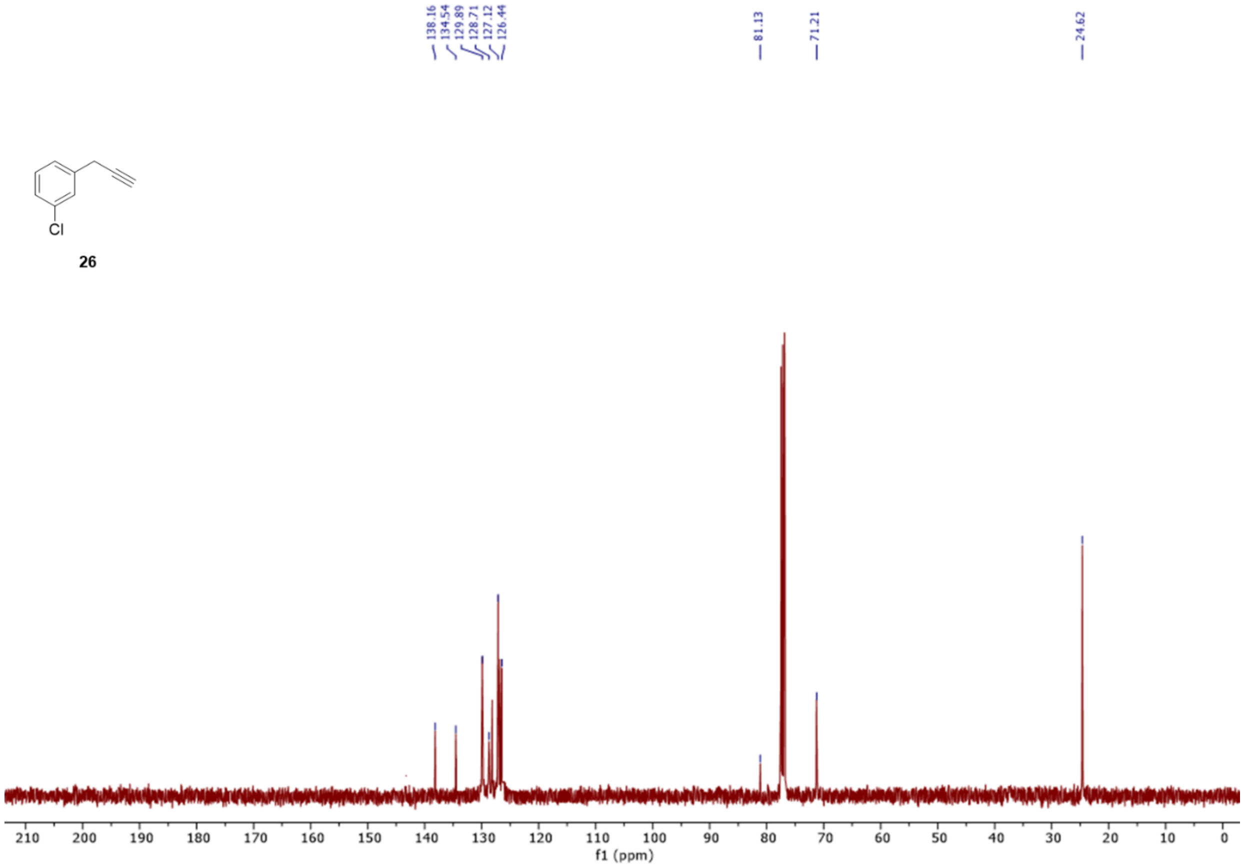

^{1}H NMR ($CDCl_3$, 400 MHz)

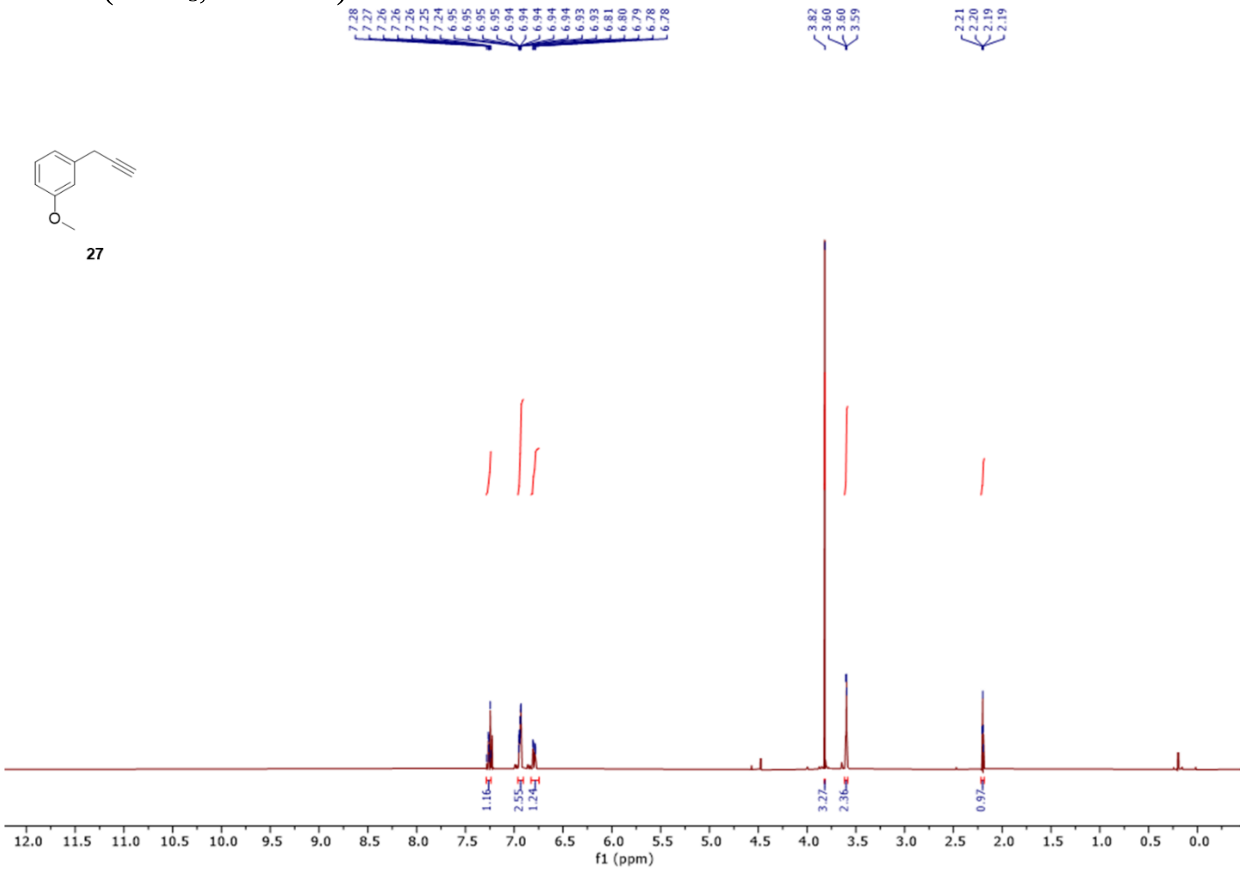

^{13}C ($CDCl_3$, 100 MHz)

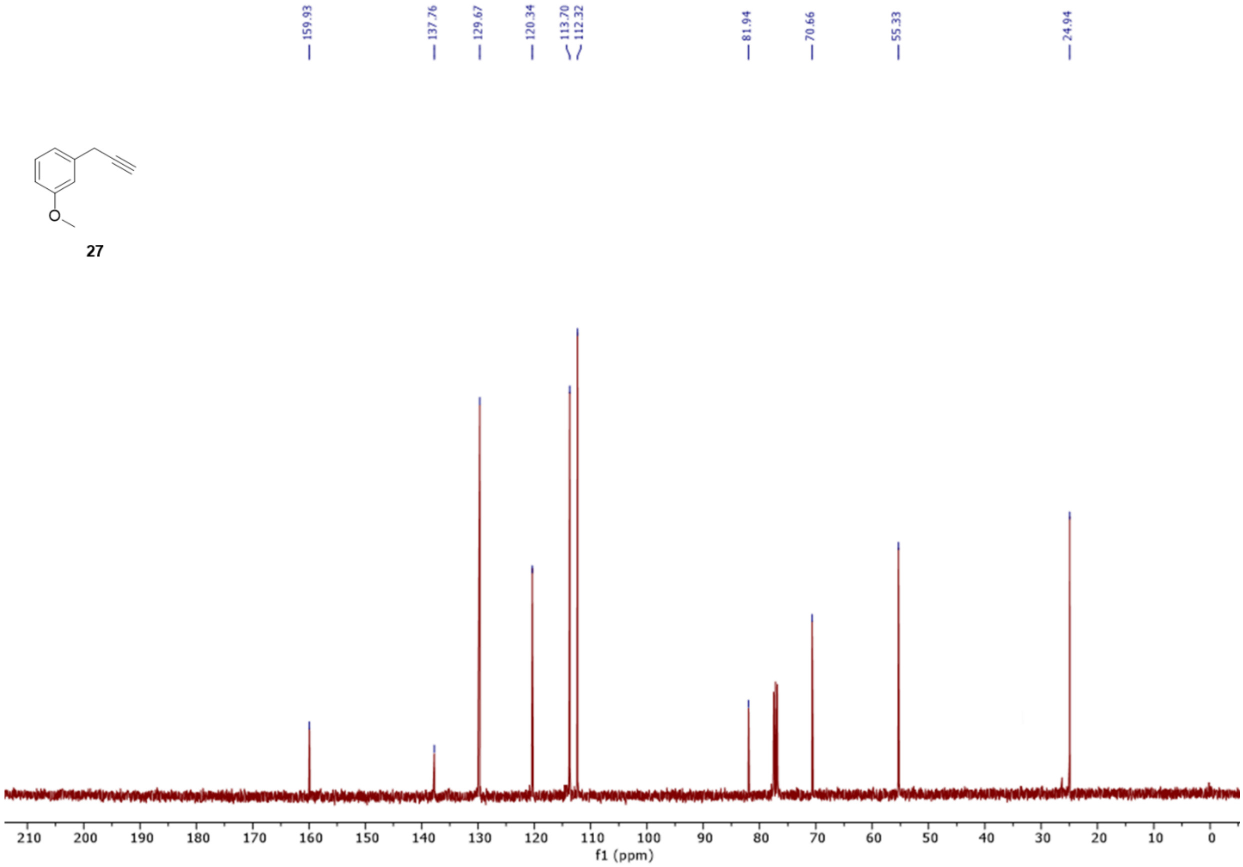

S3. NMR spectra of compounds 2-7

^{1}H NMR ($CDCl_3$, 400 MHz)

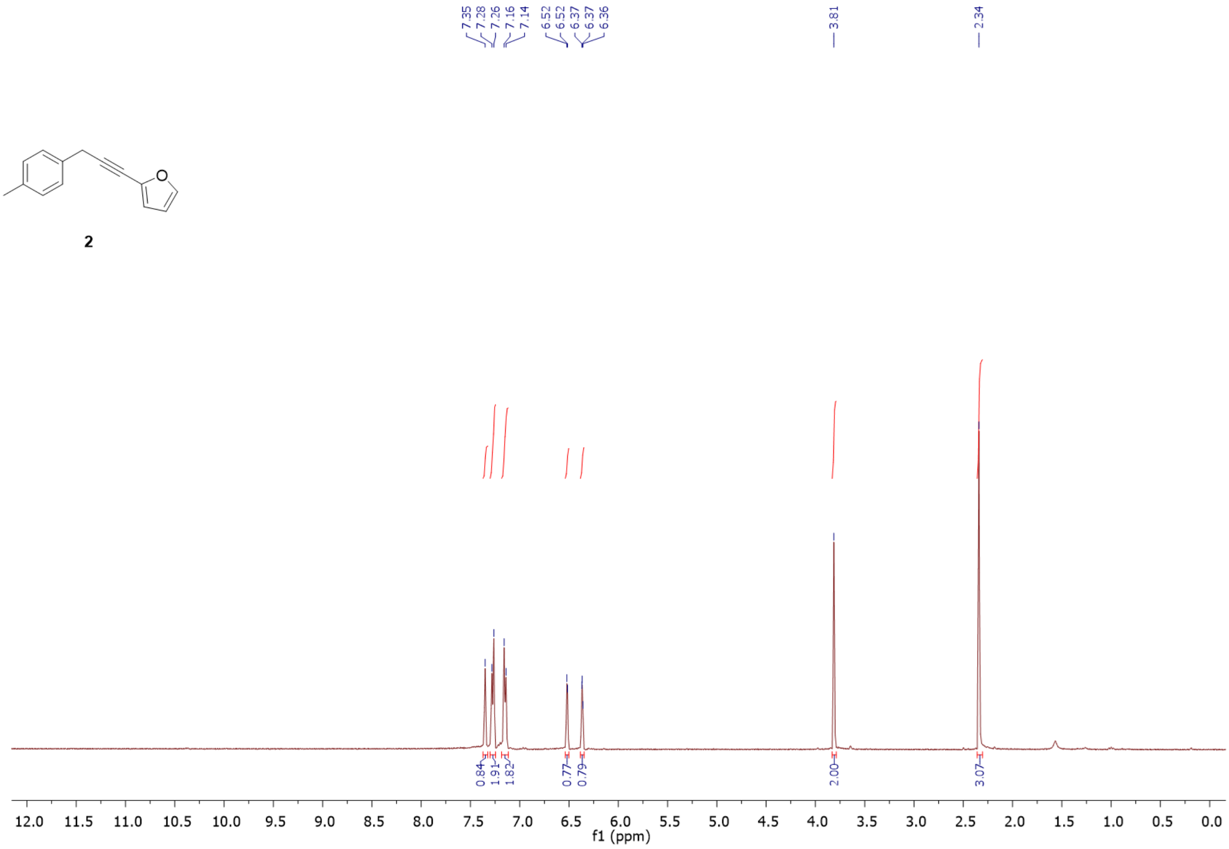

^{13}C ($CDCl_3$, 100 MHz)

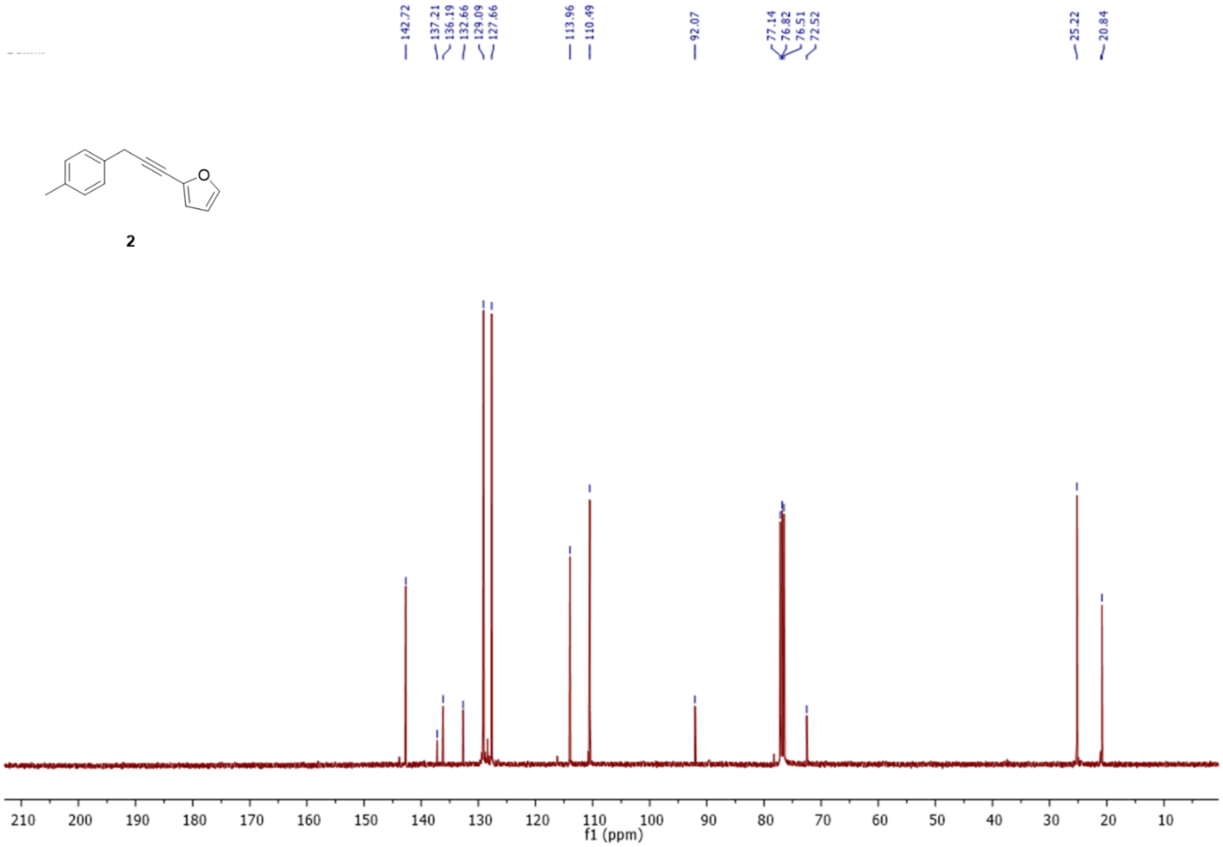

^{1}H NMR ($CDCl_3$, 500 MHz)

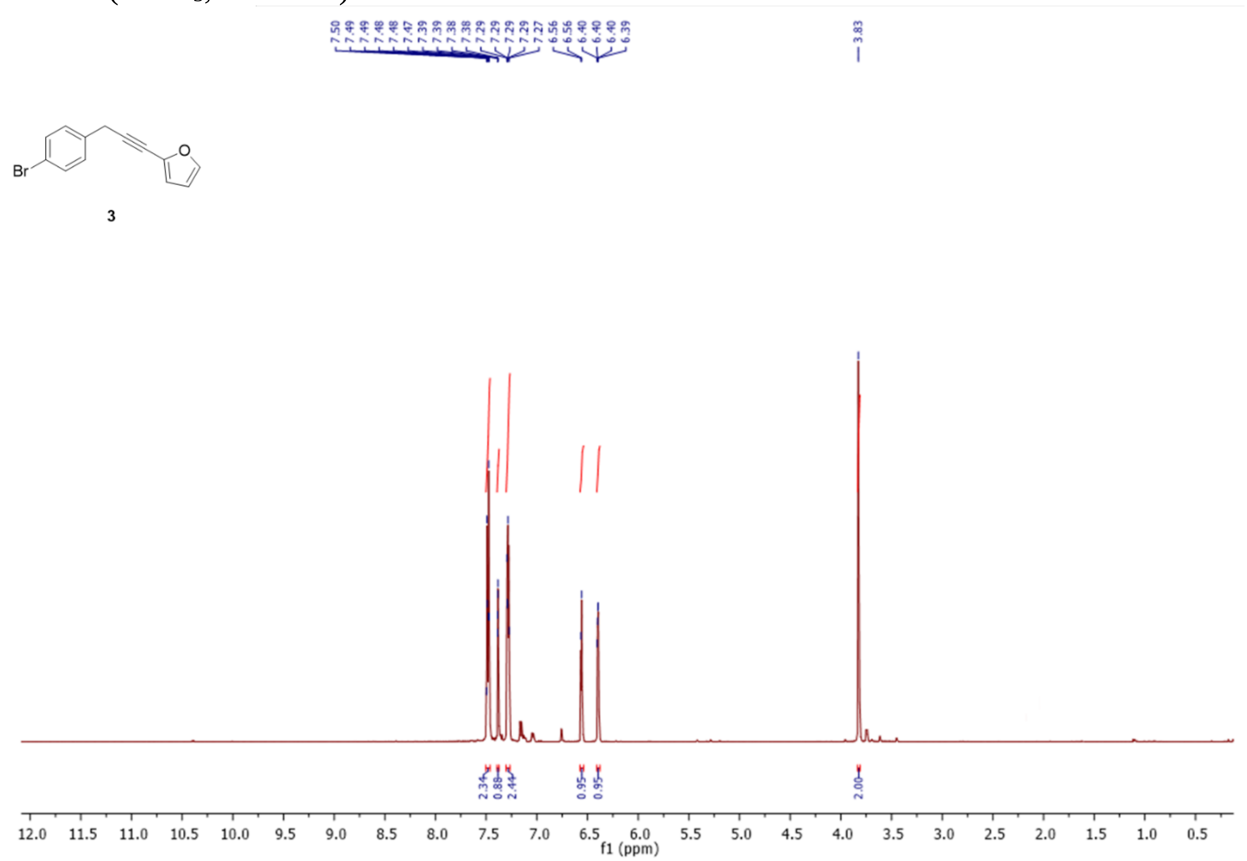

^{13}C ($CDCl_3$, 100 MHz)

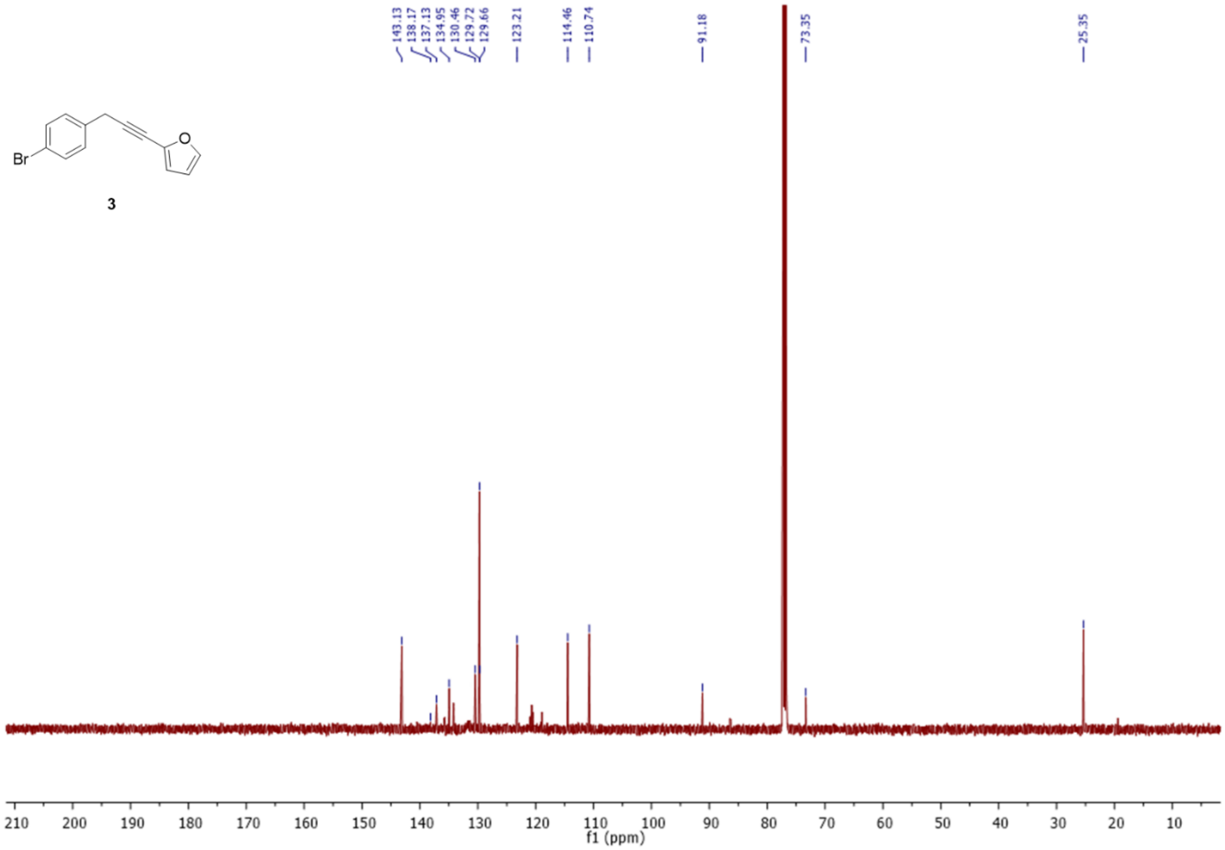

^1H NMR ($CDCl_3$, 500 MHz)

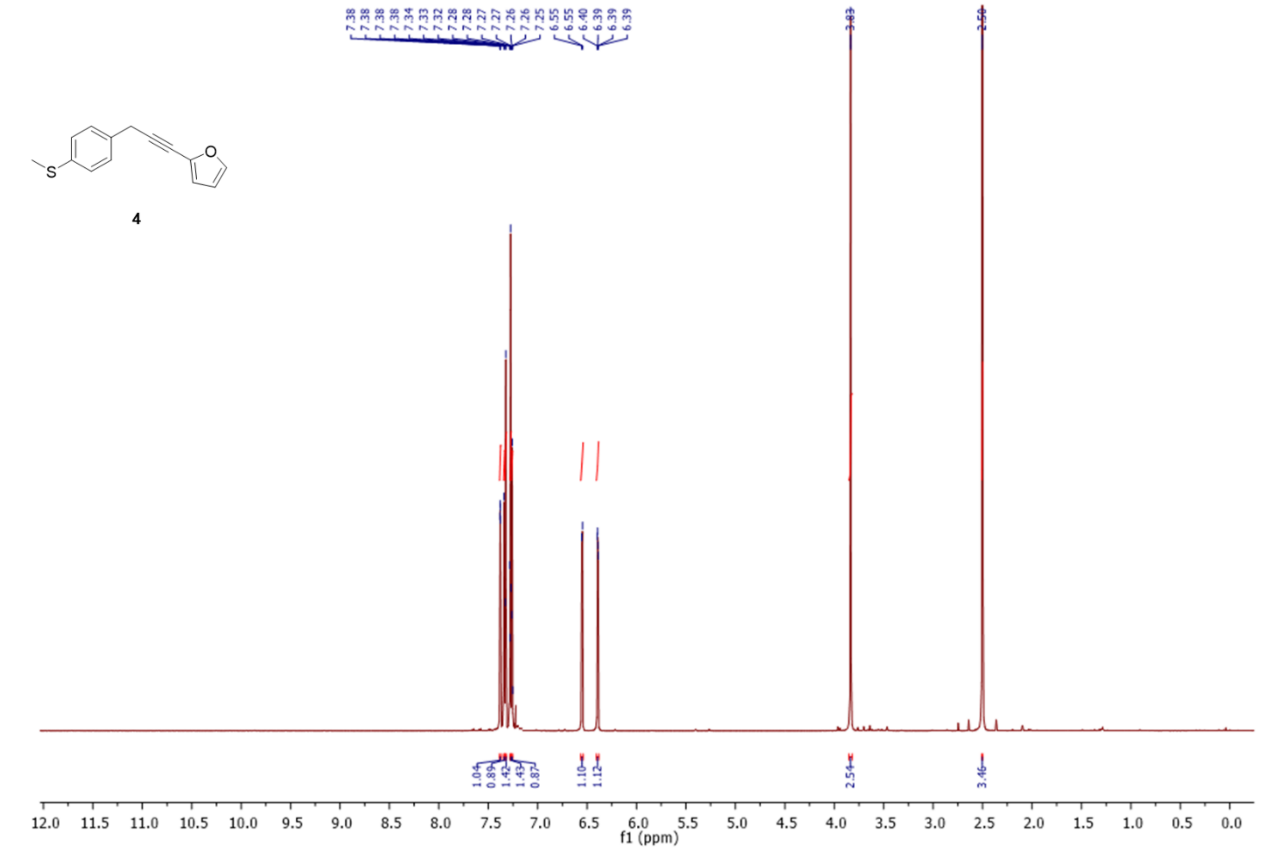

^{13}C ($CDCl_3$, 100 MHz)

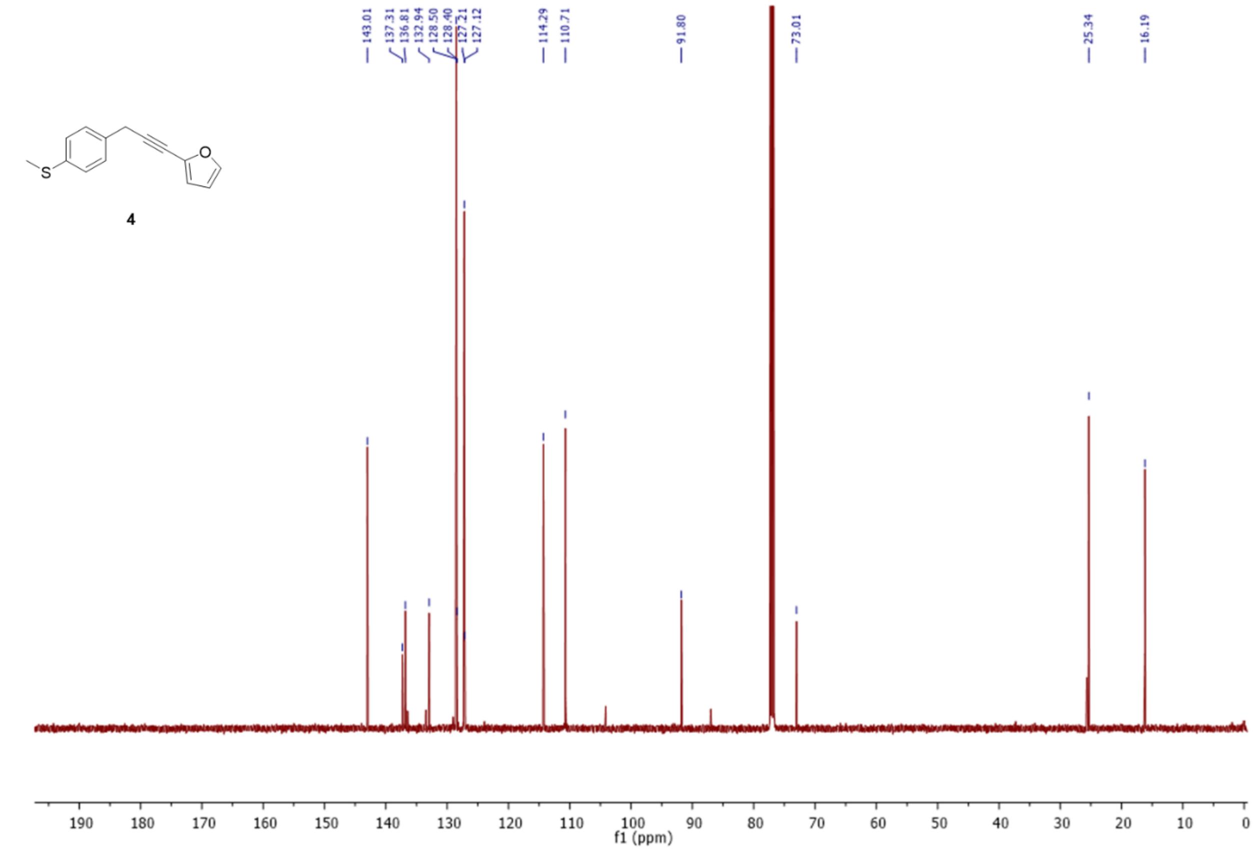

^{1}H NMR ($CDCl_3$, 400 MHz)

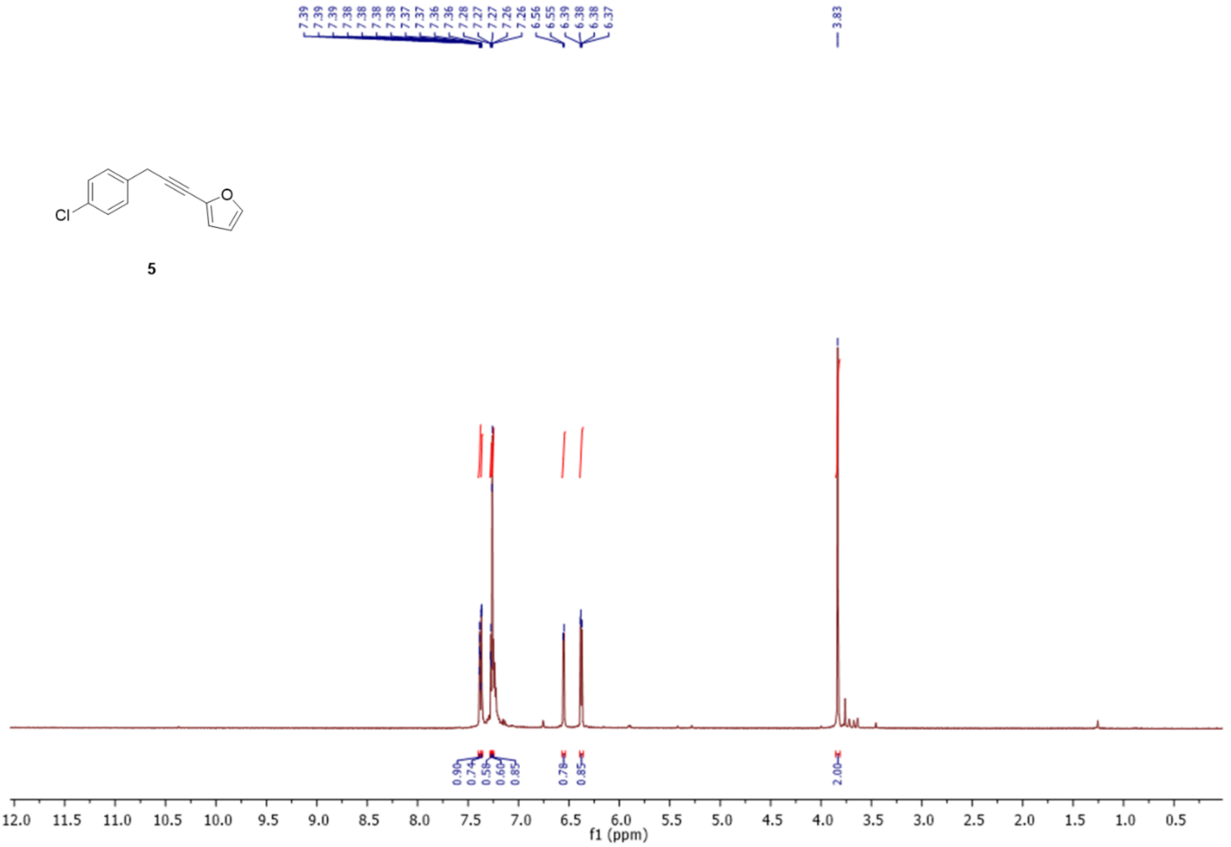

^{13}C ($CDCl_3$, 100 MHz)

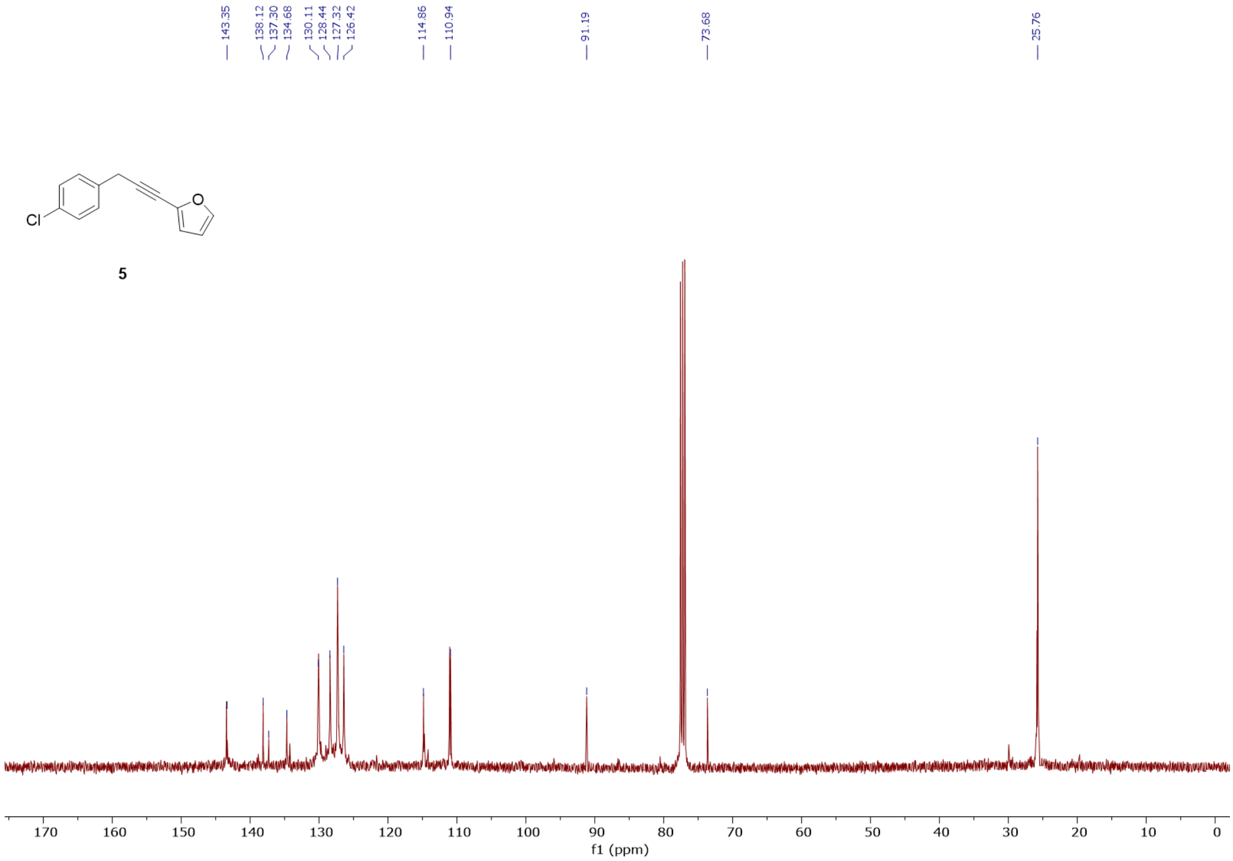

^{1}H NMR ($CDCl_3$, 400 MHz)

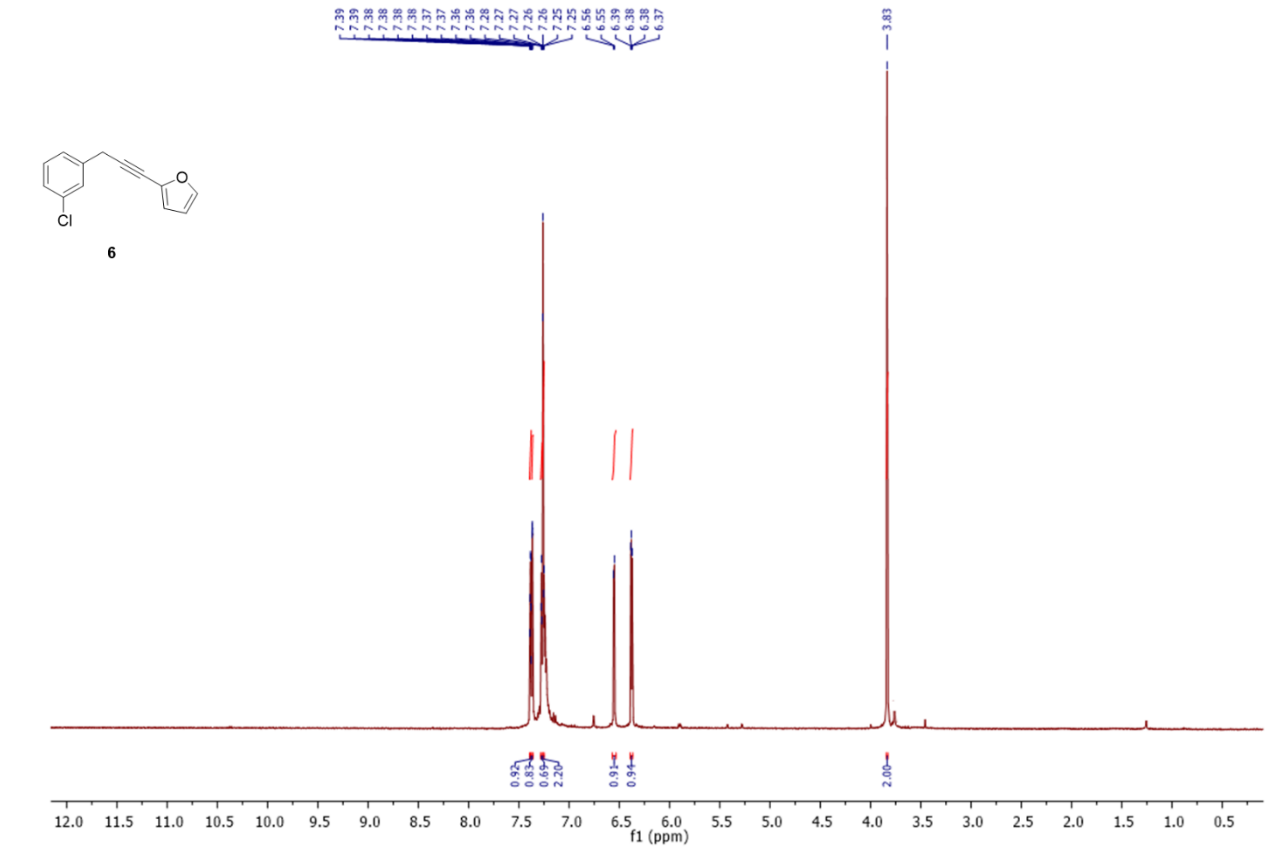

^{13}C ($CDCl_3$, 100 MHz)

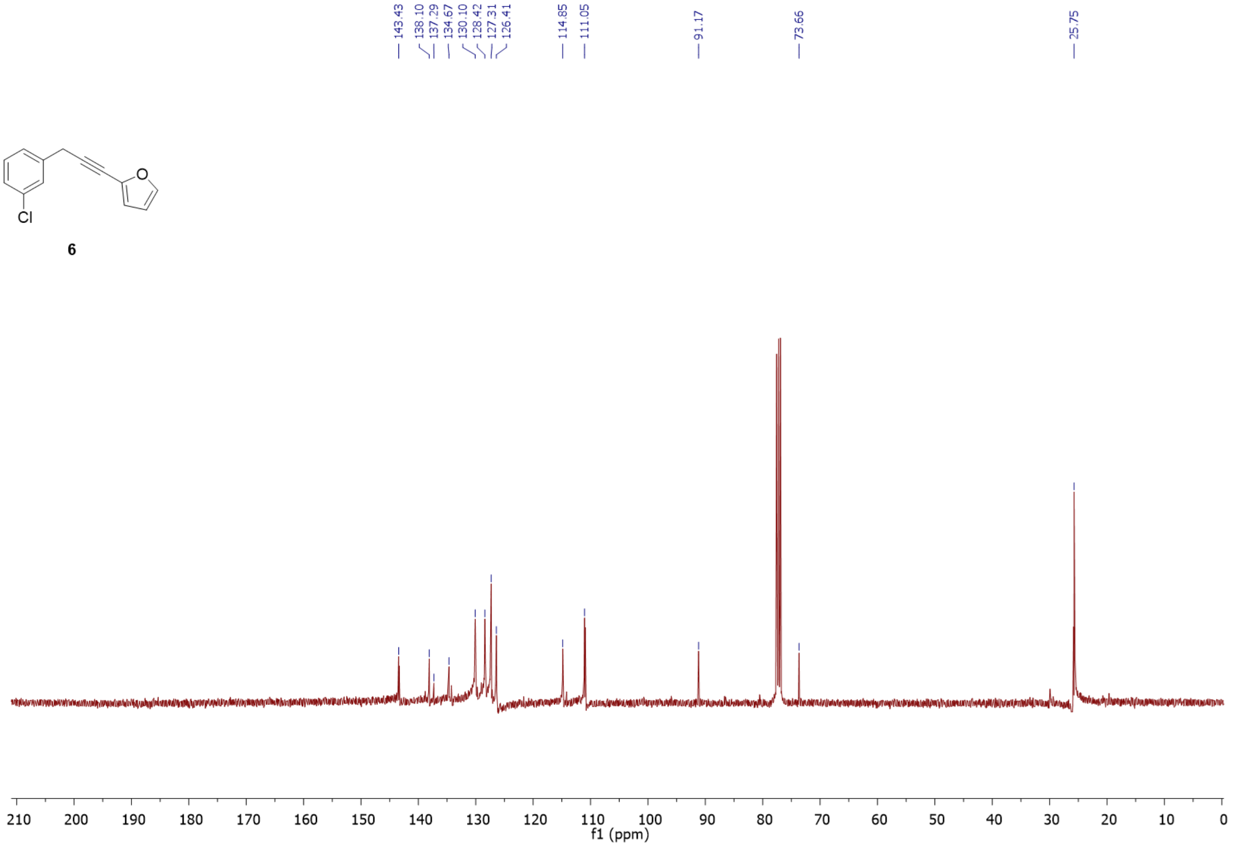

^{1}H NMR ($CDCl_3$, 400 MHz)

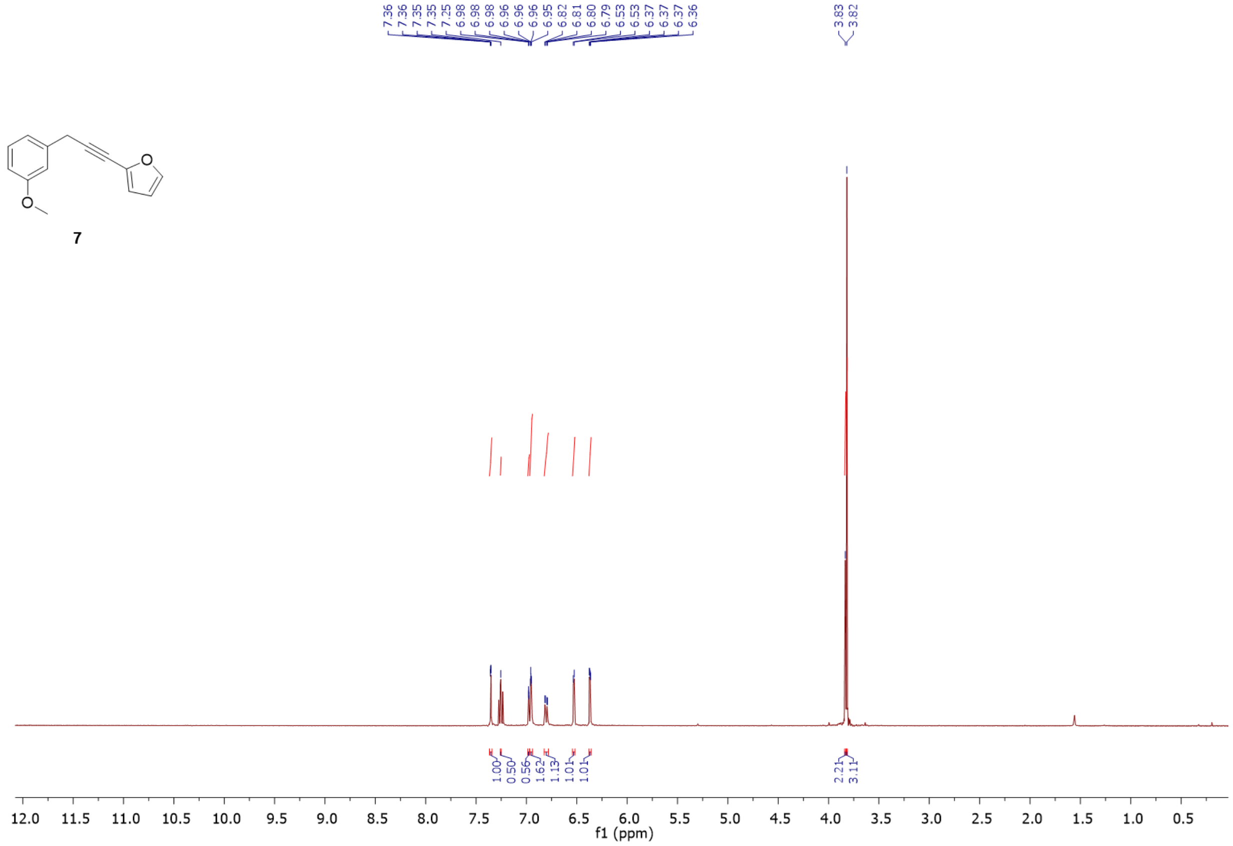

^{13}C ($CDCl_3$, 100 MHz)

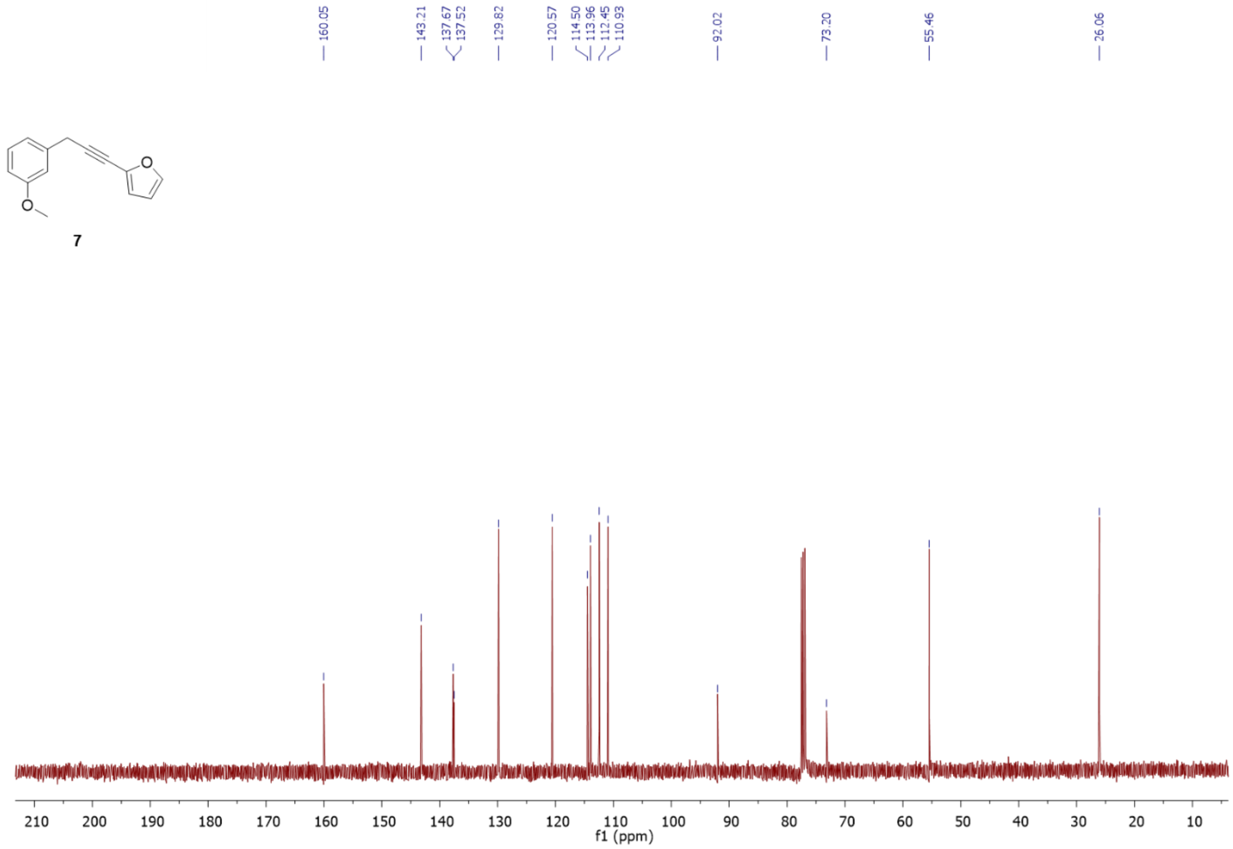

S4. GC-MS chromatogram of *Carlina acaulis* essential oil

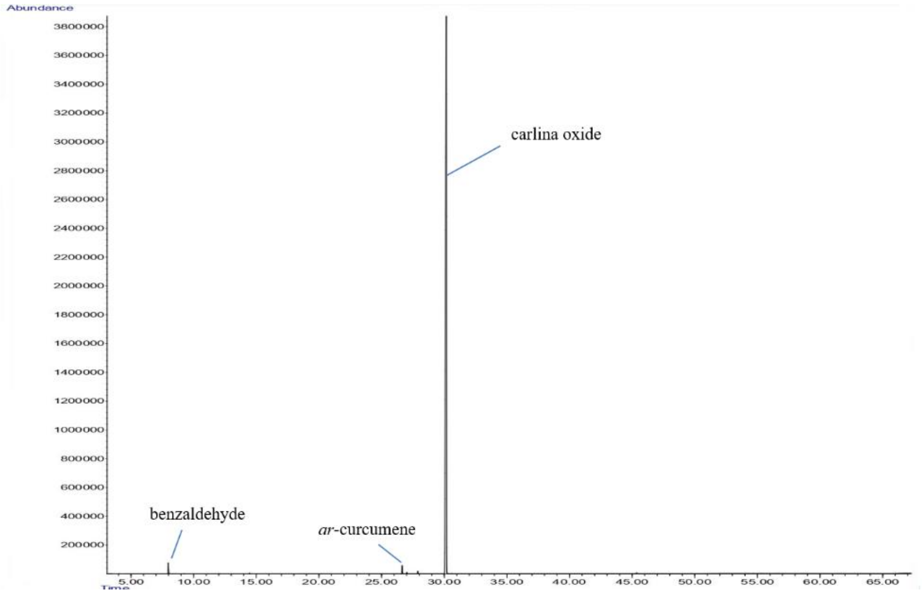

Supplement: Supplementary file 1 — np3c00137_si_001.pdf [file np3c00137_si_001.pdf]
